# Comparative Genomics Studies on the *dmrt* Gene Family in Fish

**DOI:** 10.3389/fgene.2020.563947

**Published:** 2020-11-12

**Authors:** Junjian Dong, Jia Li, Jie Hu, Chengfei Sun, Yuanyuan Tian, Wuhui Li, Ningning Yan, Chengxi Sun, Xihui Sheng, Song Yang, Qiong Shi, Xing Ye

**Affiliations:** ^1^Key Laboratory of Tropical and Subtropical Fisheries Resources Application and Cultivation, Ministry of Agriculture, Pearl River Fisheries Research Institute, Chinese Academy of Fishery Sciences, Guangzhou, China; ^2^Shenzhen Key Lab of Marine Genomics, Guangdong Provincial Key Lab of Molecular Breeding in Marine Economic Animals, BGI Academy of Marine Sciences, BGI Marine, BGI Group, Shenzhen, China; ^3^College of Fisheries, Huazhong Agricultural University, Wuhan, China; ^4^Fisheries College, Guangdong Ocean University, Zhanjiang, China; ^5^College of Animal Science and Technology, Sichuan Agricultural University, Chengdu, China

**Keywords:** fish, comparative genomics studies, *dmrt* genes, phylogenetic evolution, synteny analysis

## Abstract

Doublesex and mab-3-related transcription factor (*dmrt*) genes are widely distributed across various biological groups and play critical roles in sex determination and neural development. Here, we applied bioinformatics methods to exam cross-species changes in the dmrt family members and evolutionary relationships of the *dmrt* genes based on genomes of 17 fish species. All the examined fish species have *dmrt1–5* while only five species contained *dmrt6.* Most fish harbored two *dmrt2* paralogs (*dmrt2a* and *dmrt2b*), with *dmrt2b* being unique to fish. In the phylogenetic tree, 147 DMRT are categorized into eight groups (DMRT1–DMRT8) and then clustered in three main groups. Selective evolutionary pressure analysis indicated purifying selections on *dmrt1–3* genes and the *dmrt1–3–2(2a)* gene cluster. Similar genomic conservation patterns of the *dmrt1–dmrt3–dmrt2(2a)* gene cluster with 20-kb upstream/downstream regions in fish with various sex-determination systems were observed except for three regions with remarkable diversity. Synteny analysis revealed that *dmrt1, dmrt2a, dmrt2b*, and *dmrt3–5* were relatively conserved in fish during the evolutionary process. While *dmrt6* was lost in most species during evolution. The high conservation of the *dmrt1–dmrt3–dmrt2(2a)* gene cluster in various fish genomes suggests their crucial biological functions while various *dmrt* family members and sequences across fish species suggest different biological roles during evolution. This study provides a molecular basis for fish *dmrt* functional analysis and may serve as a reference for in-depth phylogenomics.

## Introduction

Doublesex and Mab-3-related transcription factor (*dmrt*) genes are originally homologous to *Doublesex* (*Dsx*) in *Drosophila melanogaster* and *Male abnormal 3* (*Mab-3*) in *Caenorhabditis elegans*, both of which play important roles in sex determination (Burtis and Baker, 1989; Zhu et al., 2000; Zarkower, 2001). In recent years, a large number of genes from the *dmrt* family have been identified from lower invertebrates to higher vertebrates, including corals, nematodes, fruit flies, frogs, fish, birds, and mammals, some of which have been confirmed to be related to sex differentiation (Hodgkin, 2002). Currently, in addition to *Dsx* and *Mab*, the *dmrt* family in vertebrates include nine *dmrt* genes (*dmrt1–8* and *dmrt*2b) that share common characteristics with *Dsx* and *Mab-3*. Almost all of the encoded polypeptide chains contain a conserved DNA-binding motif, known as the Doublesex and Mab-3 (DM) domain, which is composed of six conserved cysteines and two histidines (locus 1 of CCHC and locus 2 of HCCC). Both loci form two highly intertwined zinc-finger-like DNA-binding regions can bind to the minor groove in DNA. Notably, this domain is highly conserved among organisms of different evolutionary types (Erdman and Burtis, 1993; Zhu et al., 2000).

Fish *dmrt* genes were first discovered in Nile tilapia (*Oreochromis niloticus*) and rainbow trout (*Oncorhynchus mykiss*) (Guan et al., 2000; Marchand et al., 2000). These genes in the *dmrt* family have now been identified in more than 30 fish species. Seven *dmrt* genes have been found in fish, including *dmrt1*–*6* and *dmrt2b*. DMRT1 plays an important role in sex differentiation and testicular development (Matson and Zarkower, 2012), except the DM-W gene, a DMRT1 W-linked paralog in *Xenopus laevis*, play the opposite roles in primary ovary development (Yoshimoto et al., 2010). DMRT1 is specifically expressed only in the embryonic genital ridge and adult testes of human males, and is related to the expression of sex-determining genes and differentiation of primordial germ cells (Raymond et al., 1998; Moniot et al., 2000; Matson et al., 2011). Alternatively, studies on more than 20 fish species have determined that fish *dmrt1* expression is related to male development regardless of the various sex determination mechanisms (Kobayashi et al., 2004, 2008; Johnsen et al., 2010), indicating that *dmrt1* plays a key role in male germ cells self-renewal and differentiation, testicular development and spermatogenesis of fish (Herpin and Schartl, 2011; Lin et al., 2017). Furthermore, in the medaka *Oryzias latipes*, a Y-specific *dmy* gene, copy of autosome *dmrt1*, is the master sex-determining gene inducing male formation too.

The genomes of amphibians, reptiles, birds, and mammals contain only a single *dmrt2* gene, whereas fish harbor two *dmrt2* genes (*dmrt2a* and *dmrt2b*) (Liu et al., 2009; Su et al., 2015; Lyu et al., 2019). DMRT2 is widely distributed in the tissues of mammals and fish, and is expressed in both testes and ovaries (Kim et al., 2003; Winkler et al., 2004; El-Mogharbel et al., 2007). However, the function of DMRT2 has not been conserved during the evolution of species (Meng et al., 1999; Seo et al., 2006). For example, mouse DMRT2 is mainly involved in somite differentiation, in particular the patterning of the axial skeleton system (Lourenco et al., 2010). In contrast, both zebrafish *dmrt2a* and *dmrt2b* are involved in somite development, of which *dmrt2a* is necessary for symmetric somite formation and fast muscle differentiation (Saude et al., 2005; Lu et al., 2017), and *dmrt2b* regulates asymmetric organ positioning via the Hedgehog signaling pathway and therefore it is related to branchial arch and slow muscle development (Zhou et al., 2008; Li et al., 2018). This indicates that differences exist in the expression and functionality of *dmrt2a* and *dmrt2b* in fish.

Mammalian DMRT3 is highly expressed in the testis but not in the ovary; hence, it may be related to testicular differentiation and development (Hong et al., 2007). In mice, DMRT3 is also expressed in numerous non-gonadal tissues such as the embryonic forebrain and olfactory placode, in addition to spinal cord neurons, and thus it may be involved in neuronal specification (Smith et al., 2002; Kim et al., 2003; Andersson et al., 2012). Fish Dmrt3 is highly expressed in the testis and nervous system, and has accordingly been speculated to play a role in the developmental processes of the nerves and germ cells (Yamaguchi et al., 2006; Li et al., 2008; Dong et al., 2010).

The mouse *dmrt4* gene is expressed in the testis and ovary, in addition to other various tissues (Kim et al., 2003). It can regulate the formation and development of ovarian follicles (Balciuniene et al., 2006). Alternatively, *Xenopus* DMRT4 is involved in the regulation of neurogenesis in the olfactory system (Huang et al., 2005b). In some fish species, the expression of *dmrt4* in the ovary is significantly higher than that in the testis (Guan et al., 2000; Su et al., 2013; Wang, 2013); in other species, its expression is significantly higher in the testis than the ovary (Kondo et al., 2002; Dong and Chen, 2013; Sheng et al., 2014), whereas yet other species show high expression in both organs (Yamaguchi et al., 2006). In addition, *dmrt4* is also expressed in the spleen (Yamaguchi et al., 2006; Sheng et al., 2014), kidney (Kondo et al., 2002; Sheng et al., 2014), gills (Kondo et al., 2002; Wang, 2013), and brain (Dong and Chen, 2013) in fish. Hence, it has been speculated to be related to immune and nervous system development.

Mouse DMRT5 is mainly expressed in brain tissue and is necessary for the early embryonic development of the cerebral cortex (Veith et al., 2006a; Konno et al., 2012). As a novel neurogenic factor, DMRT5, together with DMRT3, jointly controls hippocampal development and neocortical area map formation (Muralidharan et al., 2017; De Clercq et al., 2018). Fish *dmrt5* is highly expressed primarily in the brain but can also be found in the gonads, eyes, and pituitary gland (Guo et al., 2004; Veith et al., 2006a; Yamaguchi et al., 2006; Gu et al., 2019). Furthermore, *dmrt5* plays a key role in zebrafish neurogenesis in the telencephalon (Yoshizawa et al., 2011) and can regulate corticotrope and gonadotrope differentiation in the pituitary (Graf et al., 2015), in addition to spermatogenesis (Xu et al., 2013).

Mammalian DMRT6 is mainly expressed in gonadal intermediate cells and differentiating spermatogonia. It plays a crucial role in coordinating the transition of primordial germ cells from the mitotic to meiotic developmental programs during spermatogenesis (Zhang X. et al., 2014) and is also expressed in the embryonic brain of mice (Kim et al., 2003). Early studies have suggested that the *dmrt6* gene is missing in fish (Veith et al., 2006b). However, recent studies have found that certain fish, such as coelacanth, tilapia, and Southern catfish also carry the *dmrt6* gene, and that tilapia *dmrt6* is involved in spermatogenesis (Forconi et al., 2013; Zhang X. et al., 2014). However, DMRT7 and DMRT8 are only present in mammals. The two genes are very similar, although DMRT8 does not have a complete DM domain. DMRT7 is specifically expressed in the male and female gonads and is related to mouse gonadal development and spermatogenesis (Kawamata and Nishimori, 2006; Hong et al., 2007). In comparison, DMRT8 is highly expressed in the male gonads and may have evolved from DMRT7 (Ottolenghi et al., 2002; Veith et al., 2006a).

Currently, reports are only available regarding the phylogenetic analysis of pan-arthropod and pan-metazoan DMRT family members (Volff et al., 2003; Wexler et al., 2014; Panara et al., 2019); to our knowledge, no studies have yet been published on the phylogeny of fish *dmrt* family. However, fish comprise a wide variety of species and previous reports have shown that members of the fish *dmrt* family own unique features such as two paralogs of *dmrt2* genes (*dmrt2a* and *dmrt2b*), in addition to diverse tissue expression of the same gene family member in various fish (e.g., *dmrt4*), thus suggesting a remarkable difference in function. As the sequences of DMRT family members are highly variable with only the DM domain [∼49 amino acids (aa)] exhibiting high sequence homology (Volff et al., 2003), it is difficult to accurately determine the evolutionary relationship among the family members based on such short sequences, which in turn has limited our understanding of the history of DMRT functional development.

Nevertheless, in recent years the whole-genome sequencing of many fish species has significantly facilitated the in-depth and systematic analysis on the evolutionary relationships among gene family members. In this study, we therefore employed the fine genomic map of largemouth bass recently obtained using third-generation sequencing by our team and collected the *dmrt* sequences of 16 fish species with different taxonomic positions from published whole-genome sequences, in order to analyze the sequence structure, phylogenetic relationship, sequence conservation, and synteny of members of the fish *dmrt* family. These findings will lay a solid foundation for a more systematic understanding of the structural characteristics of these members in fish *dmrt* family, and for further investigations into the different functions of fish *dmrt* family members in sex determination or differentiation along with their underlying mechanisms.

## Materials and Methods

### Sequence Collection

In the present study, we employed two strategies to collect nucleotide or deduced amino acid sequences for *dmrt* family members in various vertebrates (Supplementary Table S1). For those with publicly available sequences, such as in human (*Homo sapiens*) and mouse (*Mus musculus*), we downloaded the sequences from NCBI or Ensembl (Supplementary Table S2). Other *dmrt* sequences were extracted from corresponding genome databases through BLAST (Altschul et al., 1990) and Genewise (Birney et al., 2004).

In brief, we used zebrafish *(Danio rerio)*, Japanese medaka *(Oryzias latipes*), and mouse DMRT protein sequences from NCBI as the references, and mapped them onto the examined genomes using tBLASTn with an *E*-value <1e^–5^ and an alignment rate>0.6. Solar v0.9.6 was applied to connect high-identity segment pairs. Subsequently, we discarded those low-quality results with alignment rate <0.6 and mapping identity <0.5. Finally, each gene sequence was predicted on the target genomic region using Exonerate v2.2.0 (Slater and Birney, 2005), and extended 5 kb in the upstream and downstream directions to obtain the integrated gene model. A total of 147 *dmrt* sequences were derived from 23 representative vertebrate species, including 2 mammals (human and mouse), 2 birds (chicken Gallus gallus and zebra finch *Taeniopygia guttata*), 1 reptile (green Anole *Anolis carolinensis*), 1 amphibian (Western clawed frog *Xenopus tropicalis*), and 17 fish species belonging to two classes (Actinopterygii and Sarcopterygii), and ten superorders (Percomorpha: largemouth bass, Asian sea bass *Lates calcarifer*, European sea bass *Dicentrarchus labrax*, Japanese pufferfish *Takifugu rubripes*, Chinese tongue sole *Cynoglossus semilaevis*, and threespine stickleback *Gasterosteus aculeatus*; Atherinomorpha: Japanese medaka and southern platyfish *Xiphophorus maculatus*; Protacanthopterygii: Atlantic salmon *Salmo salar*; Paracanthopterygii: Atlantic cod *Gadus morhua*; Ostariophysi: channel catfish *Ictalurus punctatus* and electric eel *Electrophorus electricus*; Clupeomorpha: Atlantic herring *Clupea harengus*; Elopomorpha: Japanese eel *Anguilla japonica*; Osteoglossomorpha: Asian arowana *Scleropages formosus*; Holostei: spotted gar *Lepisosteus oculatus*; Coelacanthiformes: African coelacanth *Latimeria chalumnae*).

### Sequence Alignment and Phylogenetic Analysis

We performed phylogenetic analysis on these collected *dmrt* sequences. MAFFT v7.273 (Katoh et al., 2002) was employed to align these sequences. Gblocks was used to find conserved fragments with the following parameter settings: minimum number of sequences for a conserved/flank position (75/75), maximum number of contiguous non-conserved positions (50), minimum length of a block (50), allowed gap positions (all). ProtTest v3.42 was operated to determine the best-fit models of amino acid replacement (Darriba et al., 2011). Based on the Akaike Information Criterion (AIC) algorithm, we set the best-fit model as “JTT+I+G+F.” Finally, we utilized PhyML 3.0, MrBayes v3.24 7, and MEGA v7.0 8 to analyze these sequences with 1,000,000 generations for Ngen and 100 for Samplefreq (Ronquist et al., 2012). Branch support values were calculated using Bayesian posterior probabilities. Evolview (He et al., 2016) was applied to edit constructed phylogenetic trees.

### Identification of Conserved Synteny for the *dmrt1–dmrt3–dmrt2(2a)* Gene Cluster (Synteny Analysis)

To evaluate the conservation of the *dmrt1–dmrt3–dmrt2(2a)* gene cluster, we explored conserved genes in the upstream and downstream regions (20 kb) within the genomes of 19 examined species, using zebrafish genomic sequence as the reference, since the zebrafish genome is currently the best fish genome assembly with the highest quality and the completest genome annotation. These examined genome assemblies were explored using tBLASTn (Altschul et al., 1990), and the best-fit results were selected using a Perl script and Adobe Illustrator.

### Substitution Rate Estimation and Comparison (*Ka/Ks* Analysis)

We calculated the average non-synonymous substitutions (*Ka*), synonymous substitutions (*Ks*), and *Ka/Ks* among *dmrt1*, *dmrt2(2a)*, *dmrt3*, and the *dmrt1–dmrt3–dmrt2(2a)* gene cluster to test the selective pressure at the codon-based sequence level among various species. First, we aligned *dmrt* gene sequences from each species to spotted gar (*L. oculatus*; as the reference sequence) by using Prank v100802 with the “-codon” model (Loytynoja and Goldman, 2005). Subsequently, we calculated the *Ka*, *Ks*, and *Ka/Ks* values of each pair using *Ka/Ks* Calculator v2.0 with four different algorithms, including gMYN (Wang D.P. et al., 2009), gYN (Wang D. et al., 2009), MYN (Zhang et al., 2006), and YN (Yang and Nielsen, 2000).

### Analysis of Regulatory Regions and Cross-Species Comparisons of the *dmrt1–dmrt3–dmrt2(2a)* Gene Cluster

Complete genomic sequences with 20 kb-upstream/downstream regions of the *dmrt1–dmrt3–dmrt2(2a)* gene cluster were extracted from various species. We applied mVISTA (Frazer et al., 2004) to align these relevant genomic sequences. This tool can align and compare long sequences based on the window-based comparisons of sequence conservation.

Repetitive elements were annotated using RepeatMasker v4.06 software (Chen, 2004), and the zebrafish genomic sequence was used as the reference. Pair-wise sequence comparisons were determined with a threshold of 70% identity in each 50-bp window. In addition, five typical regulatory elements, including BRE, CAAT box, E box, GC box, and TATA box, were predicted in each sequence using a Perl script (the motif function in Primer 5.0 and Genomatix MatInspector). Finally, Adobe Illustrator and R were applied to produce graphs for the information obtained.

## Results

### Cross-Species Changes in dmrt Family Members and Copy Numbers

A total of 147 *dmrt* sequences were derived from 23 representative vertebrate species (Table 1 and Supplementary Tables S1, S2). Among them, 128 *dmrt* sequences for 17 species were downloaded from the NCBI/Ensembl databases (asterisk in Table 1 and Accession number in Supplementary Table S2). The remaining 19 *dmrt* sequences for three species were extracted from genomes through the method described in section “Similarities and Variances of the *dmrt* Gene Family Members in Various Fish Species.” These nucleotide sequences and corresponding deduced protein sequences were used for our further data analysis.

**TABLE 1:** Identification of the *dmrt* family genes in the examined vertebrates.

Class	Superorder	Species	Common name	*dmrt1*	*dmrt2* (*dmrt2a/b*)	*dmrt3*	*dmrt4* (*dmrtA1*)	*dmrt5* (*dmrtA2*)	*dmrt6* (*dmrtB1*)	*dmrt7* (*dmrtC2*)	*dmrt8* (*dmrtC1*)
				
				Numbers							
Mammalian	–	*H. sapiens*	Human	1*	1*	1*	1*	1*	1*	1*	1*
	–	*M. musculus*	Mouse	1*	1*	1*	1*	1*	1*	1*	1*
Birds	–	*G. gallus*	Chicken	1*	1*	1*	-	1*	1*	-	-
	–	*T. guttata*	Zebra finch	1*	1*	1*	-	1*	1*	-	-
Reptilia	–	*A. carolinensis*	Anole lizard	1*	1*	1*	1*	1*	1*	-	-
Amphibia	–	*X. tropicalis*	Clawed frog	1*	1*	1*	1*	1*	1*	-	-
Fish (Actinopterygii)	Percomorpha	*M. salmoides*	Largemouth bass	1	2	1	1	1	1	-	-
		*L. calcarifer*	Asian seabass	1*	2*	1*	1*	1*	1*	-	-
		*D. labrax*	European seabass	1	2	1	1	1	-	-	-
		*T. rubripes*	Japanese pufferfish	1*	2*	1*	1*	1*	-	-	-
		*C. semilaevis*	Tongue sole	1*	2*	1*	1*	1*	-	-	-
		*G. aculeatus*	Stickleback	1*	2*	1*	1*	1*	-	-	-
	Atherinomorpha	*O. latipes*	Japanese medaka	1*	2*	1*	1*	1*	-	-	-
		*X. maculatus*	Southern platyfish	1*	2*	1*	1*	1*	-	-	-
	Protacanthopterygii	*S. salar*	Atlantic salmon	1*	4*	2*	1*	2*	-	-	-
	Paracanthopterygii	*G. morhua*	Atlantic cod	1*	1*	1*	1*	1*	-	-	-
	Ostariophysi	*D. rerio*	Zebrafish	1*	2*	1*	-	1*	-	-	-
		*I. punctatus*	Channel catfish	1*	2*	1*	1*	1*	1*	-	-
		*E. electricus*	Electronic eel	1	2	1	1	1	-	-	-
	Clupeomorpha	*C. harengus*	Atlantic herring	1*	2*	1*	1*	1*	-	-	-
	Elopomorpha	*A. japonica*	Japanese eel	1*	1*	2*	1*	1*	-	-	-
	Osteoglossomorpha	*S. formosus*	Asian arowana	1*	2*	1*	1*	1*	-	-	-
	Holostei	*L. oculatus*	Spotted gar	1*	2*	1*	1*	1*	1*	-	-
Fish (Sarcopterygii)	Coelacanthiformes	*L. chalumnae*	Coelacanth	1*	1*	1*	1*	1*	1*	-	-

*Asterisks indicate the dmrt genes were downloaded from the NCBI/Ensembl databases.*

In mammals, eight *dmrt* genes (*dmrt1*–*dmrt8*) were identified in their genomes. However, in other species, *dmrt7* and *dmrt8* were lost. In addition, *dmrt4* was also lost in birds. In the fish *dmrt* gene family, *dmrt1*–*dmrt5* showed relatively high conservation. Among these, *dmrt2* usually consisted of two paralogs (*dmrt2a* and *dmrt2b*) in most fish species, with only three species (Atlantic cod, Japanese eel, and coelacanth) carrying a single paralog. *dmrt6* was only found in five fish species, i.e., largemouth bass, Asian sea bass, channel catfish, spotted gar, and African coelacanth. In addition, some of the *dmrt* genes were duplicated in Atlantic salmon (*dmrt2*, *3*, *5*) and Japanese eel (*dmrt3*; see Table 1).

### Structural Characterization and Evolutionary Analysis of the *dmrt* Family Genes

**FIGURE 1:**
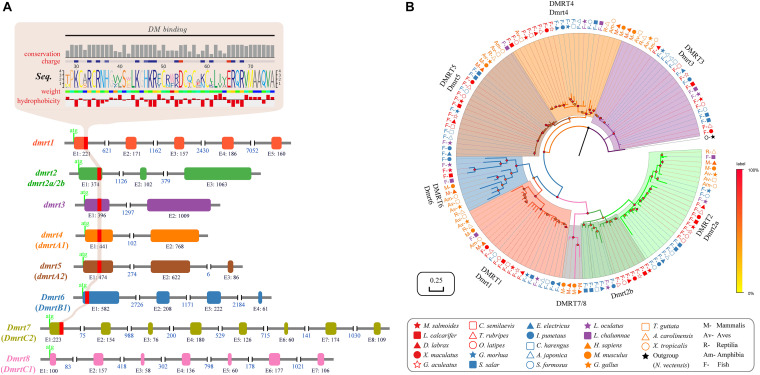
Characterization and phylogenetic analysis of the *dmrt* family in vertebrates. **(A)** Various structures of *dmrt* genes. The genomic structures of *dmrt1–dmrt5* are based on the data of Japanese pufferfish, whereas those of *dmrt6–dmrt8* are derived from the relevant data of mouse. **(B)** A Bayesian phylogenetic tree of 147 *dmrt* sequences. The phylogenetic analysis was performed using MrBayes v3.2.6. Amino acid replacement model selection was calculated using ProtTest with the best-fit model of JTT+I+G+F. The tree is rooted with the *N. vectensis dmrtA*.

The gene structure of *dmrt1* is composed of five exons in all examined species except for Atlantic salmon (Table 2 and Figure 1A), and a highly conserved DM domain (with a total of 49 aa) is located in the DMRT1 protein. In comparison, *dmrt2* contains three exons and *dmrt3*–*dmrt4* contain two exons in most examined species. *Dmrt5* consists of 2 to 4 exons in higher vertebrates but only two in all examined fish species except for Stickleback (Table 2). *Dmrt6* contains four exons in higher vertebrates, whereas the number of exons in fish varies greatly (from 2 to 4). *dmrt7* and *dmrt8* can only be identified in mammals, and both contain a large number of exons (8 for *dmrt7*, 6–7 for *dmrt8*). Except for DMRT8, all DMRT proteins (DMRT1–7) contain a conserved DM domain, often locating in the first exon of each gene (Figure 1A).

**TABLE 2:** Exon numbers in *dmrt* family genes of the examined vertebrates.

Class	Superorder	Species	Common name	*dmrt1*	*dmrt2* (*dmrt2a/b*)	*dmrt3*	*dmrt4* (*dmrtA1*)	*dmrt5* (*dmrtA2*)	*dmrt6* (*dmrtB1*)	*dmrt7* (*dmrtC2*)	*dmrt8* (*dmrtC1*)
				
				Exon numbers							
Mammalian	–	*H. sapiens*	Human	5	3	2	2	3	4	8	6
	–	*M. musculus*	Mouse	5	3	2	2	3	4	8	7
Birds	–	*G. gallus*	Chicken	5	3	2	-	2	4	-	-
	–	*T. guttata*	Zebra finch	5	3	2	-	2	4	-	-
Reptilia	–	*A. carolinensis*	Anole lizard	5	3	2	2	4	4	-	-
Amphibia	–	*X. tropicalis*	Clawed frog	5	3	2	2	3	4	-	-
Fish (Actinopterygii)	Percomorpha	*M. salmoides*	Largemouth bass	5	3/3	2	2	2	3	-	-
		*L. calcarifer*	Asian seabass	5	3/3	2	2	2	3	-	-
		*D. labrax*	European seabass	5	3/3	2	2	2	-	-	-
		*T. rubripes*	Japanese pufferfish	5	3/3	2	2	2	-	-	-
		*C. semilaevis*	Tongue sole	5	4/3	2	2	2	-	-	-
		*G. aculeatus*	Stickleback	5	4/3	5	3	3	-	-	-
	Atherinomorpha	*O. latipes*	Japanese medaka	5	4/3	2	2	2	-	-	-
		*X. maculatus*	Southern platyfish	5	4/3	2	2	2	-	-	-
	Protacanthopterygii	*S. salar*	Atlantic salmon	3	4/4/3/3	2/1	2	2/2	-	-	-
	Paracanthopterygii	*G. morhua*	Atlantic cod	5	3	2	2	2	-	-	-
	Ostariophysi	*D. rerio*	Zebrafish	5	3/3	2	-	2	-	-	-
		*I. punctatus*	Channel catfish	5	3/3	2	2	2	4	-	-
		*E. electricus*	Electronic eel	5	3/3	2	2	2	-	-	-
	Clupeomorpha	*C. harengus*	Atlantic herring	5	3/3	2	2	2	-	-	-
	Elopomorpha	*A. japonica*	Japanese eel	5	3	2/2	2	2	-	-	-
	Osteoglossomorpha	*S. formosus*	Asian arowana	5	4/3	2	2	2	-	-	-
	Holostei	*L. oculatus*	Spotted gar	5	3/3	2	2	2	2	-	-
Fish (Sarcopterygii)	Coelacanthiformes	*L. chalumnae*	Coelacanth	5	3	2	2	2	4	-	-

Using DMRTA protein sequence of the sea anemone (*Nematostella vectensis*) as the out-group, we constructed a protein-based phylogenetic tree (Figure 1B), in which the DMRT family is distinctly categorized into eight groups (DMRT1–DMRT8). All DMRT proteins are distributed in the following three main groups: Group 1 includes five subfamilies, i.e., DMRT1, 2, 6, 7, and 8. DMRT2 was placed as the sister of DMRT7/8 and DMRT1 as the sister of DMRT6, suggesting a closer evolutionary relationship among these subfamilies. The subfamilies DMRT2, 7, and 8 were together placed as a sister group to the DMRT1 and 6 subfamilies. Group 2 includes DMRT4 and DMRT5. Group 3 contains only one subfamily DMRT3 (see more details in Figure 1B).

### The *dmrt1-dmrt3-dmrt2(2a)* Cluster in Fish Genomes

**FIGURE 2:**
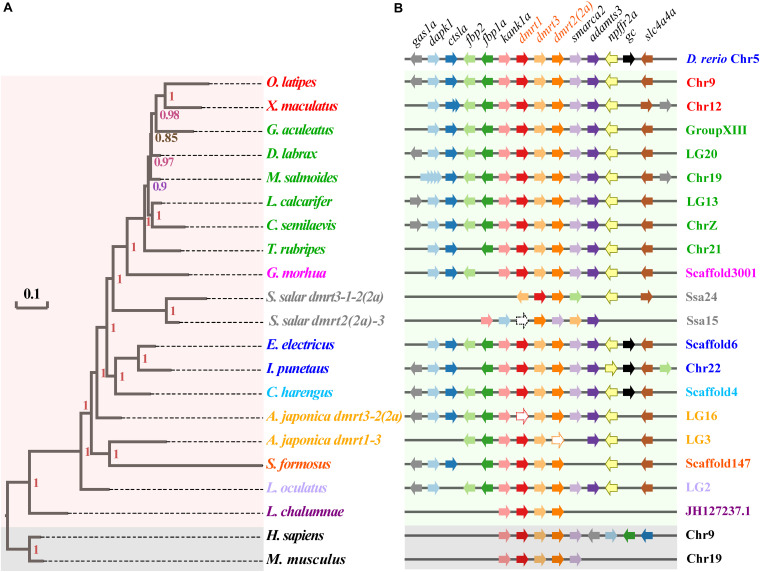
Phylogenetic tree and synteny conservation of the fish *dmrt1-dmrt3-dmrt2(2a)* gene clusters. **(A)** The rectangular Bayesian phylogenetic tree, rooted with human and mouse sequences. Numbers besides the nodes are Bayesian posterior probabilities (colored). **(B)** The synteny of examined *dmrt1-dmrt3-dmrt2(2a)* gene clusters. Full names of relevant genes are provided in Supplementary Table S3.

Syntenic relationships of the *dmrt1-dmrt3-dmrt2(2a)* gene cluster were analyzed in 17 fish species, and using zebrafish (Cypriniformes, Ostariophysi) as the base reference. The *dmrt1–dmrt3–dmrt2(2a)* gene cluster is relatively conserved in various fish species; consistent with this, fish within the same superorder were clustered together (Figure 2A). A total of 11 genes (*gas1a, dapk1, ctsla, fbp2, fbp1a, kank1a, smarca2, adamts3, npffr2a, gc*, and *slc4a4a*) neighbor the zebrafish *dmrt1–dmrt3–dmrt2(2a)* gene cluster (Supplementary Table S3). All these genes were also found to neighbor the *dmrt1–dmrt3–dmrt2(2a)* gene cluster in channel catfish and Atlantic herring, whereas some genes were lost around this gene cluster in the remainder fish species (with frequent loss of the *gc*).

Mammals have lost larger numbers of genes next to this cluster, which also happens in two fish species (Atlantic salmon and coelacanth). Furthermore, in some fish species, such as largemouth bass, the *dapk1* gene experienced a polyploidization event to generate four tandem duplicated copies. This phenomenon was also observed in the southern platyfish, which harbors two copies of the *ctsla* gene in its genome. Moreover, in both largemouth bass and southern platyfish, the *gas1a* gene experienced a translocation and inversion event as well (see more details in Figure 2B).

### Substitution Rates (*Ka/Ks*) of the *dmrt1–dmrt3–dmrt2(2a)* Cluster in Fish Genomes

*Ka/Ks* represents the ratio of non-synonymous substitutions (*Ka*) to synonymous substitutions (*Ks*). This ratio can be used to determine whether there is selective pressure on a given protein-coding gene. It is generally believed that synonymous mutations are not subjected to natural selection, whereas non-synonymous mutations are. *Ka/Ks* > 1 implies the existence of positive selection; *Ka/Ks* = 1 suggests neutral selection; and *Ka/K*s < 1 indicates purifying selection.

**FIGURE 3:**
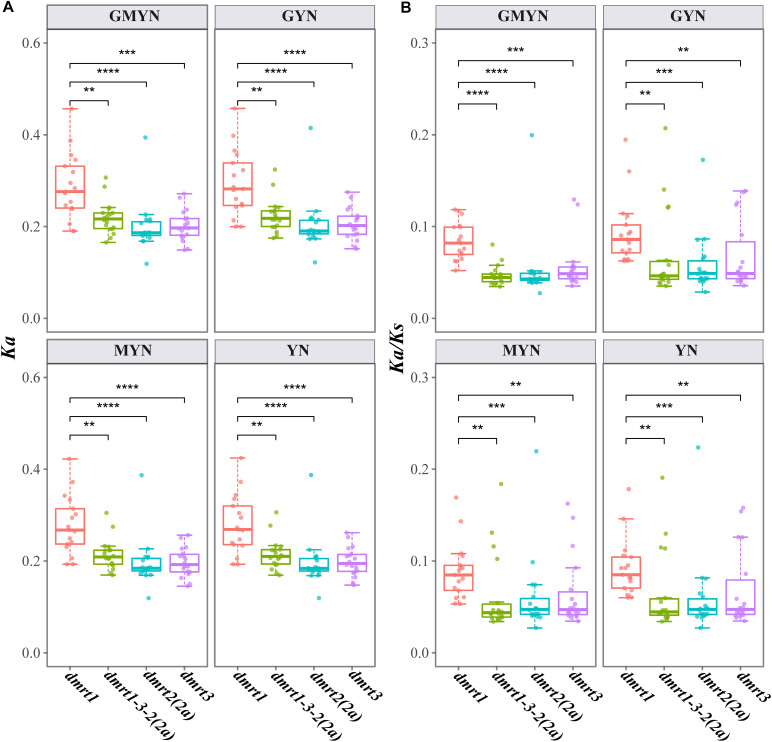
*Ka* **(A)** and *Ka/Ks* **(B)** values for the target coding sequences. Paralog genes in the spotted gar were used as the references. **p < 0.01; ***p < 0.001; and ****p < 0.0001 (based on a Wilcoxon test). Four methods, including GMYN, GYN, MYN, and YN, were used.

Comparing the *Ka* and *Ka/Ks* values using four different methods (gMYN, gYN, MYN, and YN), we found conserved substitution rates in *dmrt1*, *dmrt2(2a)*, *dmrt3*, and the *dmrt1–dmrt3–dmrt2(2a)* gene cluster. In detail, all the mean *Ka*/*Ks* values were less than 1 (Figure 3), indicating a purifying selection on these genes. However, *dmrt1* showed a higher average *Ka/Ks* value (0.0900) than *dmrt2* (0.0576), *dmrt3* (0.0645), and the *dmrt1–dmrt3–dmrt2(2a)* gene cluster (0.0606), suggesting that the evolution of *dmrt1* might be less conservative and thereby may provide more variants for selection.

### Conserved Sequences and Regulatory Elements in the Fish *dmrt1–dmrt3–dmrt2(2a)* Gene Clusters

**FIGURE 4:**
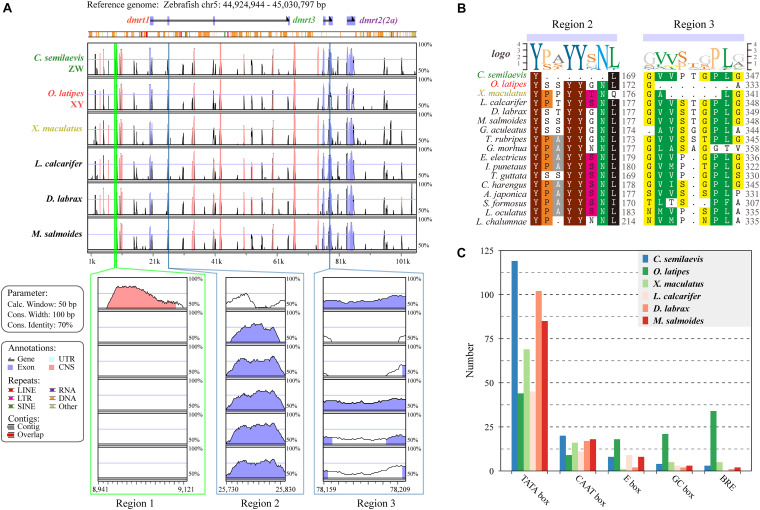
Species variances of the regulatory regions in the *dmrt1-dmrt3-dmrt2(2a)* gene cluster. **(A)** A VISTA plot of the *dmrt1-dmrt3-dmrt2(2a)* gene clusters among six examined fish species. Peaks of similarity in pair-wise sequence alignments between zebrafish (*D. rerio*) are compared with Chinese tongue sole (*C. semilaevis*), Japanese medaka (*O. latipes*), southern platyfish (*X. maculatus*), Asian sea bass (*L. calcarifer*), European sea bass (*D. labrax*), and largemouth bass (*M. salmoides*). Blue peaks represent coding exons and pink peaks denote non-coding sequences. The horizontal axis shows relative positions in the zebrafish genomic sequence, whereas the vertical axis indicates percentage of identity (50–100%). Three distinct regions, named as Region 1, Region 2, and Region 3, were identified. **(B)** Protein sequence alignments of the Regions 2 and 3 in 17 examined fish species. **(C)** Numbers of different regulatory elements in six representative fish species. Additional details regarding the sequences of each regulatory element are provided in Supplementary Table S4.

To visualize the genomic conservation, the mVISTA tool was employed to generate a VISTA plot (Figure 4A). We used Chinese tongue sole (ZZ/ZW) (Chen et al., 2014), Japanese medaka (XX/XY) (Kasahara et al., 2007), southern platyfish (males are XY or YY, females are WX, WY, or XX) (Volff and Schartl, 2001), European sea bass (polygenic sex determination system, PSD system) (Vandeputte et al., 2007; Mei and Gui, 2015), Asia sea bass (protandrous hermaphrodite) (Wang et al., 2018), and largemouth bass which was previously reported as WZ/ZZ) (Glennon et al., 2012), whereas our recent analysis combining genomic map, ddRAD-Seq and sex-reversal experiments suggests a XX/XY sex determination system (data unpublished),, to evaluate regulatory elements and genomic sequence changes in the *dmrt1–dmrt3–dmrt2(2a)* gene cluster with 20-kb upstream/downstream regions in various sex-determination systems.

Overall, a similar conservation pattern in both coding and non-coding sequences was observed. Comparisons of these six fish species along with zebrafish showed considerable homology within and between these *dmrt* genes. We also identified three regions with remarkable diversity among these fish (lower panels in Figure 4A). Region 1 covers 207 bp located at the 11-kb upstream region of the *dmrt1* gene and contains nine TATA boxes (63 bp), which only exists in Chinese tongue sole. Region 2 is located in the third exon of *dmrt1* with 18-bp missing in Chinese tongue sole. Comparing the protein sequences of tongue sole and other fish species, we determined that six amino acids (-P/S-A/S/T/P-YY-S/G/N-N-) were missing (Figure 4B). Region 3, located in the second exon of *dmrt3*, shows a 21-nucleotide (nt) deletion in Japanese medaka and a 15-nt deletion in southern platfish (Figure 4B).

In the examined six species, TATA box represents the main regulatory element. In Chinese tongue sole, TATA boxes are much more frequent than in other fish, however, markedly fewer E boxes, GC boxes, and B recognition elements (BREs) are present in Japanese medaka than in other species (Figure 4C).

### Synteny of Other *dmrt* Genes [Excluding *dmrt1, dmrt2(2a)*, and *dmrt3*] in Fish Genomes

**FIGURE 5:**
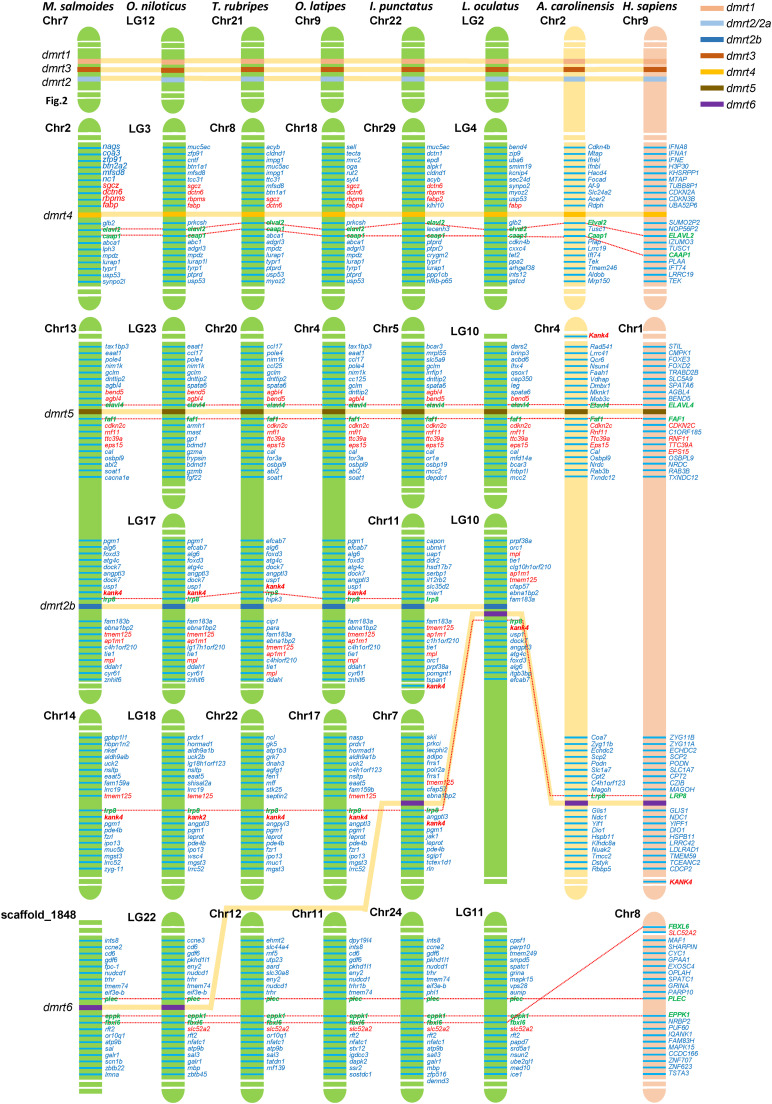
Synteny of *dmrt* genes in various fish species. The synteny analyses were performed in eight vertebrate species (*M. salmoides*, *O. niloticus*, *T. rubripes*, *O. latipes*, *I. punctatus*, *L. oculatus*, *A. carolinensis*, and *H. sapiens*). Chr., chromosome; LG, linkage group. The most conserved surrounding genes of *dmrt* genes were shown in green and bold fonts. The most conserved were shown in red fonts.

Based on the whole-genome sequence of largemouth bass and other eight representative vertebrate species (including *O. niloticus*, *T. rubripes*, *O. latipes*, *I. punctatus*, *L. oculatus*, *A. carolinensis*, and *H. sapiens*) obtained from NCBI, we performed a synteny analysis of four *dmrt* genes, including *dmrt2b*, *dmrt4*, *dmrt5*, and *dmrt6*. The results (Figure 5) indicated that among these fish species, the KN motif and ankyrin repeat domain-containing protein 4 (*kank4*) and low-density lipoprotein receptor-related protein 8 (*lrp8*) genes in the upstream of *dmrt2b* were conserved. The ELAV-like protein 2 (*elavl2*) and caspase activity and apoptosis inhibitor 1 (*caap1*) genes in the downstream of *dmrt4*, and the *elavl4* and FAS-associated factor 1 (*faf1*) genes in the downstream of *dmrt5* were also conserved, which is consistent with the findings in reptiles and humans. This suggests that *dmrt2b*, *dmrt4*, and *dmrt5* were relatively conserved during the evolutionary process.

Although *dmrt6* was lost in most fish species including *T. rubripes* and *O. latipes*, in *L. oculatus* and *I. punctatus*, *lrp8* was present in the upstream of *dmrt6*, which is consistent with higher vertebrates; whereas in *M. salmoides* and *O. niloticus*, *dmrt6* was located between the conserved plectin (*plec*) and epiplakin-F-box/LRR-repeat protein 6 (*eppk1*–*fbxl6*) genes (see more details in Figure 5).

## Discussion

Fish are the oldest and most diverse group among vertebrates, containing about 32,000 species and accounting for more than half of the vertebrate species. Fish have undergone a long history of emergence, development, and evolution. The increasing amount of fish genomic information provides an important resource for studying the evolution, structure, and function of key genes through comparative genomics analysis. Seven *dmrt* genes have been identified in fish to date, including *dmrt1–6* and *dmrt2b*. *dmrt* genes have also been reported in more than 30 fish species and a number of functional studies have been performed to reveal that regardless of the sex determination mechanism, the majority of fish *dmrt* genes (Table 3) are related to sexual development (Li et al., 2008, 2018; Liu et al., 2009; Herpin and Schartl, 2011; Yoshizawa et al., 2011; Xu et al., 2013). However, the phylogenetics of the *dmrt* gene family in fish have not yet been reported.

**TABLE 3:** The relevant references for *dmrt* family genes in fish.

Genes	Number of species	References
*dmrt1*	23	*Acipenser gueldenstaedtii* (Fajkowska et al., 2016); *A. japonica* (Jeng et al., 2019); *Acanthopagrus schlegelii* (Wu and Chang, 2018); *Anoplopoma fimbria* (Smith et al., 2013); *C. semilaevis* (Cui et al., 2017); *D. rerio* (Lin et al., 2017; Webster et al., 2017); *Epinephelus coioides* (Lyu et al., 2019); *G. morhua* (Johnsen and Andersen, 2012); *Gobiocypris rarus* (Cao et al., 2012); *Halichoeres poecilopterus* (Miyake et al., 2012); *L. chalumnae* (Forconi et al., 2013); *M. amblycephala* (Su et al., 2015); *M. salmoides* (Yan et al., 2019); *O. latipes* and *X. maculatus* (Kondo et al., 2002); *O. niloticus* (Wei et al., 2019); *Odontesthes bonariensis* (Fernandino et al., 2008); *O. mykiss* (Marchand et al., 2000); *Plecoglossus altivelis* (Wang et al., 2014); *Sebastes schlegeli* (Ma et al., 2014); *Solea senegalensis* (Portela-Bens et al., 2017); *T. rubripes* (Yamaguchi et al., 2006); *X. maculatus* (Veith et al., 2006b)
*dmrt2 (dmrt2, 2a)*	15	*C. semilaevis* (Zhu et al., 2019); *Carassius auratus* (Jiang et al., 2012); *Carassius auratus gibelio* (Liu and Gui, 2011); *D. rerio* (Zhou et al., 2008; Lu et al., 2017); *G. morhua* (Johnsen and Andersen, 2012); *E. coioides* (Lyu et al., 2019); *Labeo rohita* (Sahoo et al., 2019); *M. albus* (Sheng et al., 2014); *M. amblycephala* (Su et al., 2015); *O. latipes* and *X. maculatus* (Kondo et al., 2002); *S. senegalensis* (Portela-Bens et al., 2017); *Scophthalmus maximus* (Robledo et al., 2015); *T. rubripes* (Xu et al., 2013); *X. maculatus* (Veith et al., 2006b)
*dmrt2b*	5	*Carassius auratus* (Jiang et al., 2012); *Carassius auratus gibelio* (Liu and Gui, 2011); *D. rerio* (Li et al., 2018); *E. coioides* (Lyu et al., 2019); *G. morhua* (Johnsen and Andersen, 2012); *M. albus* (Sheng et al., 2014); *M. amblycephala* (Su et al., 2015); *T. rubripes* (Yamaguchi et al., 2006)
*dmrt3*	10	*D. rerio* (Li et al., 2008); *E. coioides* (Lyu et al., 2019); *G. morhua* (Johnsen and Andersen, 2012); *L. chalumnae* (Forconi et al., 2013); *M. albus* (Sheng et al., 2014); *M. amblycephala* (Su et al., 2015); *O. latipes* and *X. maculatus* (Kondo et al., 2002); *S. senegalensis* (Portela-Bens et al., 2017); *T. rubripes* (Yamaguchi et al., 2006)
*dmrt4 (dmrta1)*	11	*C. semilaevis* (Dong and Chen, 2013); *G. morhua* (Johnsen and Andersen, 2012); *M. albus* (Sheng et al., 2014); *M. amblycephala* (Su et al., 2013); *O. aureus* (Cao et al., 2007); *O. latipes* and *X. maculatus* (Kondo et al., 2002); *Paralichthys olivaceus* (Wen et al., 2009); *S. senegalensis* (Portela-Bens et al., 2017); *T. rubripes* (Yamaguchi et al., 2006); *X. maculatus* (Veith et al., 2006b)
*dmrt5 (dmrta2)*	7	*D. rerio* (Guo et al., 2004); *G. morhua* (Johnsen and Andersen, 2012); *M. albus* (Sheng et al., 2014); *O. niloticus* (Shirak et al., 2006); *P. altivelis* (Wang et al., 2014); *Scatophagus argus* (Gu et al., 2019); *T. rubripes* (Yamaguchi et al., 2006); *X. maculatus* (Veith et al., 2006b)
*dmrt6 (dmrtb1)*	2	*L. chalumnae* (Forconi et al., 2013); *O. niloticus* (Zhang X. et al., 2014)

To obtain a better understanding of the functional diversification of this gene family, we therefore examined dmrt gene complements from the whole genome sequences of 17 representative fish species representing 10 various superorders and several non-fish outgroups. The evolutionary relationships of the dmrt genes in fish were subsequently examined using both phylogenetic and synteny analyses.

### Similarities and Variances of the *dmrt* Gene Family Members in Various Fish Species

Zhou et al. (2008) showed that unlike mammals and other groups that only harbored one *dmrt2*, zebrafish carries a second paralog of *dmrt2(2a)*, *dmrt2b*, which was subsequently identified in many other fish species (Zhou et al., 2008; Liu et al., 2009; Su et al., 2015; Lyu et al., 2019). The 17 representative fish species analyzed in the present study belong to Actinopterygii, with the exception of coelacanth *L. chalumnae* that belongs to Sarcopterygii. Among the 16 actinopterygians, 14 harbored the two paralogs of *dmrt2* (*dmrt2a* and *dmrt2b*), however, *dmrt6*, which is commonly found in mammals and other groups, was only identified in four actinopterygians including *M. salmoides* and the sarcopterygian *L. chalumnae*.

Among the 17 fish species, only *A. japonica* and *G. morhua* carried *dmrt2a* alone and lacked *dmrt6*. *L. chalumnae* only had one *dmrt2* (*2a*) and one *dmrt6*, similar to higher vertebrates. A search through the database revealed that two other sarcopterygians (*Protopterus annectens* and *Latimeria menadoensis*) also only carried one *dmrt2a* and *dmrt6* (see more details in Supplementary Table S2; Forconi et al., 2013; Biscotti et al., 2018). Actinopterygii and Sarcopterygii are two relatively independent evolutionary branches of fish. Sarcopterygii is a side-branch in the evolution of fish, from which tetrapods evolved (Nelson et al., 2016). Therefore, the characteristics of the *dmrt* family genes in Sarcopterygii are more similar to those of higher vertebrates.

Based on the cross-species comparisons of *dmrt* family genes and copy numbers, we found that some of the *dmrt* genes were duplicated in *S. salar* and *A. japonica* (*S. salar*: *dmrt*2, 3, 5; *A. japonica*: *dmrt1*–*3*; see Table 1). Lien et al. (2016) suggested that *S. salar* is a typical tetraploid teleost that had experienced a salmonid-specific genome duplication. The copies of *dmrt* genes were duplicated in its genome, whereas one copy of *dmrt1* and *dmrt4* were lost (Lien et al., 2016). Loss of the duplicated gene possibly occurred owing to the salmonid-specific genome duplication event, which may lead to rearrangements of genome sequences, as *S. salar* has lost numerous syntenic genes in comparison with other teleosts. Similar *dmrt* duplication and loss were also found in four other fish species (e.g., brown trout *Salmo trutta* and Sockeye salmon *Oncorhynchus nerka*) that belong to the same superorder as *S. salar* (i.e., Protacanthopterygii; Supplementary Table S5). In addition, the copy number of *dmrt1* and *dmrt3* is doubled in *A. japonica*, which is considered to be an uncommon ploidy (2n = 38) of this special teleost (Nomura et al., 2004).

The conservation of fish *dmrt1* and *dmrt(3–5)* sequences is relatively high, all of which containing the highly conserved DM domain and a stable number of exons (majority of *dmrt1* contained 5 exons and most *dmrt3–5* had 2 exons). Phylogenetic analysis showed that *dmrt4* and *5* were clustered into a major branch, indicating that these genes appear to be originated from a common ancestor of *dmrt*.

To date, the *dmrt7* and *dmrt8* genes have not been found in fish but only in mammals. In fact, they exist in all mammals, from the lower Monotremata in Prototheria (platypus) (Tsend-Ayush et al., 2009) to Marsupiala in Metatheria (wombat), and to the higher Euarchonta in Eutheria (mouse) (Veith et al., 2006a), thus indicating that both genes were only formed after the evolutionary divergence of mammals from other vertebrates including fish (Veith et al., 2006a).

Apart from *dmrt5*, which is highly expressed in brain tissue, the other members of the fish *dmrt* family are highly expressed in the gonads. Specifically, *dmrt1*, *3*, and *6* are highly expressed in the testis (*dmrt1*: (Guan et al., 2000; Marchand et al., 2000; He et al., 2003; Guo et al., 2005; Huang et al., 2005a; Veith et al., 2006b; Yamaguchi et al., 2006; Johnsen et al., 2010; Su et al., 2015); *dmrt3*:(Yamaguchi et al., 2006; Dong et al., 2010; Sheng et al., 2014; Su et al., 2015); *dmrt6*: (Forconi et al., 2013; Zhang X. et al., 2014), whereas *dmrt2a*, *2b*, and *dmrt4* are expressed in both male and female gonads, with different fish species showing different expression profiles [*dmrt2a/b*:(Yamaguchi et al., 2006; Zhou et al., 2008; Liu and Gui, 2011; Sheng et al., 2014; Su et al., 2015); *dmrt4*:(Kondo et al., 2002; Veith et al., 2006b; Yamaguchi et al., 2006; Cao et al., 2007; Wen et al., 2009; Dong and Chen, 2013; Sheng et al., 2014; Jiang et al., 2019)]. Current studies have shown that fish *dmrt* family members may mainly be involved in embryonic sex differentiation, gonadal development, and gametogenesis (Herpin and Schartl, 2011; Xu et al., 2013; Zhang X. et al., 2014; Graf et al., 2015), in addition to other functions such as neural development (Li et al., 2008; Lourenco et al., 2010; Yoshizawa et al., 2011).

### Similarities and Variances of the *dmrt1–dmrt3–dmrt2(2a)* Gene Cluster in Various Fish Genomes

In vertebrate genomes, the *dmrt1*, *dmrt2(2a)*, and *dmrt3* genes are in tandem in the order of *dmrt1–dmrt3–dmrt2(2a)* (Johnsen and Andersen, 2012). Our phylogenetic analysis based on this *dmrt1–dmrt3–dmrt2(2a)* cluster confirmed the clustering in fish within the same superorder, thus indicating that the *dmrt1–dmrt3–dmrt2(2a)* gene cluster is highly conserved in various fish species (Figure 2). Further analysis of the conserved genes flanking this cluster revealed that *D. rerio* carried 11 neighboring genes, as did *I. punctatus* and *C. harengus*. However, other fish species showed partial loss (such as the *gc* gene), duplication (*dapk1* in *M. salmoides* and *ctsla* in *X. maculatus*), and transversion (*gas1a* in *M. salmoides* and *X. maculatus*). This may have been caused by genomic polyploidization events during the evolutionary process of fish (Braasch and Postlethwait, 2012). Despite the large variations in the flanking genes among different fish species, the number and location of the *dmrt1–dmrt3–dmrt2(2a)* genes have been stable. Thus, the high conservation of the *dmrt1–dmrt3–dmrt2(2a)* gene cluster in various fish genomes suggests their crucial biological functions in fish.

Among the fish genomes analyzed in this study, the *Ka/Ks* ratios of the *dmrt1–dmrt3–dmrt2(2a) gene* cluster and the three *dmrt* genes were less than 0.2, impling that after the examined actinopterygians diverged from *L. oculatus*, the *dmrt1–dmrt3–dmrt2(2a)* gene cluster was subjected to relatively strong purification selection in its evolutionary process, whereas its positive selection may have occurred prior to the divergence from *L. oculatus*. These low *Ka/Ks* ratios across various fish species indicate that the *dmrt1–dmrt3–dmrt2(2a)* genes are highly conserved during evolution. Occurrence of a non-synonymous substitution would alter the conformation and function of the corresponding protein, thereby affecting any individual’s sex differentiation, which in turn would affect the inheritance of the mutation site by its offspring (Wang D. et al., 2009). Therefore, the high conservation of the *dmrt1/2/3* genes across fish suggests that its key role in sex differentiation.

Analysis of the conserved sequences and regulatory elements was performed on the *dmrt1*–*dmrt3*–*dmrt2(2a)* gene cluster of three representative fish genomes with different sex determination systems [i.e., *C. semilaevis* (ZW) (Chen et al., 2012), *O. latipes* (XY) (Otake et al., 2006), *X. maculatus* (WXY) (Schultheis et al., 2009)]. Three distinct regions 1–3 were identified (Figure 4A). *C. semilaevis* showed 207-bp only exists in Region 1 and 18-bp deletions in Regions 2, respectively, and Region 1 contained nine TATA boxes. *O. latipes* and *X. maculatus* showed 21- and 15-bp deletions, respectively, in Region 3. Analysis of the regulatory elements for this gene cluster indicated that the number of TATA boxes in *C. semilaevis* was higher than that in other fish species (twice of *O. latipes*), whereas *O. latipes* had significantly more E box, GC box, and BRE elements than other fish species. Fish with various sex-determination systems showed significant differences in their conserved sequences and regulatory elements, suggesting that the *dmrt1–dmrt3–dmrt2(2a)* gene cluster may be related to the sex-determination systems in fish. In our recent study, it reveals that *M. salmoides* is a XY/XX system species (Sun et al., 2020). In conserved sequences analysis of fish *dmrt1–dmrt3–dmrt2(2a)* gene clusters, it had much difference in Regions 1–3 between *M. salmoides* and *C. semilaevis* (ZW/ZZ). The Region 2 of *M. salmoides* was more similar to *O. latipes* (XX/XY). The Region 3 of *M. salmoides* was similar to *D. labrax* (PSD) and *L. calcarifer* (hermaphrodite) (Wang et al., 2018). Therefore, the sex-determination systems of *M. salmoides* might be preferred to XY/XX system species.

### Conserved Synteny of the *dmrt* Genes in Fish Genomes

The synteny analysis performed in this study showed that apart from *dmrt6*, all other six *dmrt* genes in the fish *dmrt* gene family (*dmrt1*, *dmrt2a*, *dmrt2b*, and *dmrt3–5*) were relatively conserved. Fish *dmrt1–dmrt3–dmrt2(2a)* clusters are located in tandem in genomes, which is consistent with higher vertebrates. Fish *dmrt4* is usually located on a different chromosome from the cluster, and the downstream *elavl2* gene is conserved. In contrast, *dmrt4* is located on the same chromosome as this *dmrt1–dmrt3–dmrt2(2a)* cluster in higher vertebrates, but the downstream *elavl2* gene is also conserved. Fish *dmrt5* gene is the same as that in higher vertebrates, in which the upstream and downstream *elavl4* and *faf1* genes are also conserved. *dmrt2b* gene can be found in most fish species, and the upstream *kank4* and *lrp8* genes are conserved. The *dmrt6* gene is lost in most fish genomes. However, in *L. oculatus* and *I. punctatus*, *dmrt6* is conserved with downstream *lrp8*, which is consistent with higher vertebrates, however, in *M. salmoides* and *O. niloticus*, the *dmrt6* gene is located between the conserved *plec* and *eppk1*–*fbxl6* genes (see Figure 5).

**FIGURE 6:**
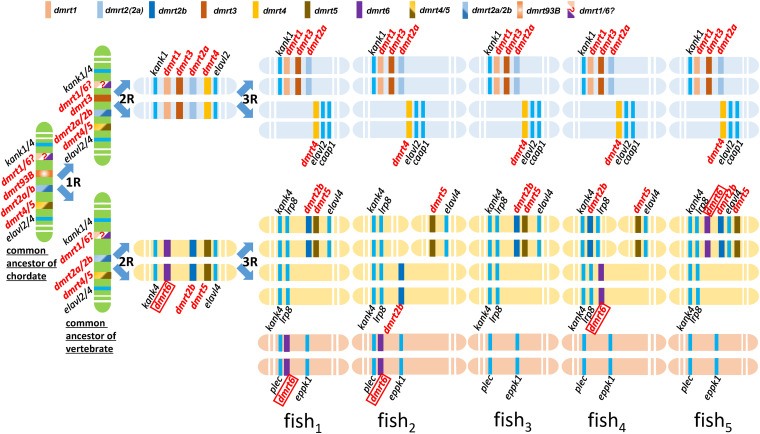
Hypothetical evolutionary history of the *dmrt* genes in fish through fish-specific (3R) genome duplications. This figure was constructed based on figures in this study. The common ancestor of chordate might possess four ancestral genes, including *dmrt4*/*5*, *dmrt2a*/*2b*, *dmrt93B*, and *dmrt1*/*6*. A common ancestor of vertebrata may have possessed four *dmrt* family genes, *dmrt1*/*6*, *dmrt2a*/*2b*, *dmrt3*, and *dmrt4*/*5*. The syntenies of *kank1-dmrt1-dmrt3-dmrt2a*, *dmrt4-elavl2-caap1*, *kank4-lrp8-dmrt2b*, and *dmrt5*- *elavl4* are conserved after three rounds of whole genome duplication in the ancestral vertebrates. *dmrt6* is lost in most fish species.

Kondo et al. (2002) was the first report of conserved synteny analysis on the *dmrt1–4* genes between fish and human. This study demonstrated that the *dmrt1*, *2*, and *3* genes formed clusters in fish and constituted a part of a large number of genes in this cluster that exhibit conserved synteny between human and fish. Johnsen and Andersen (2012) performed chromosomal synteny analysis on *dmrt2a* and *dmrt2b*, and proposed that these genes originated from the second round (2R) of whole genome duplication of the ancestral *dmrt2* (Johnsen and Andersen, 2012). In turn, Mawaribuchi et al. (2019) performed phylogenetic cluster analysis of lower bilaterian and higher animal *dmrt* genes, based on which they speculated that the *dmrt3* gene emerged by genome duplication (1R), and *dmrt1* and *dmrt6* emerged after the 2R genome duplication; they also proposed an evolutionary history for the *dmrt* family genes in bilateria (Mawaribuchi et al., 2019). Therefore, according to our data coupled with these relevant literatures, we hypothesized evolutionary history of the *dmrt* genes in fish (Figure 6).

Firstly, we propose that *dmrt* genes exist in various fish species through the fish-specific (3R) genome duplications (Johnsen and Andersen, 2012). Secondly, we propose that *dmrt3* might originate from *dmrt93B* and emerged by genome duplication (1R). According to our phylogenic tree of the *dmrt* family in vertebrates (Figure 1B), in addition to the conserved genes flanking *dmrt1* and *dmrt6* (*kank1/4*) (Figure 5), we propose that *dmrt1* and *dmrt6* might originate from the same ancestral *dmrt* (labeled with dmrt1/6? in Figure 6) and they emerged after genome duplication (2R). Thirdly, (1) we propose that the common ancestor of chordates might have four ancestral *dmrt* genes (*dmrt4/5*, *dmrt2a/2b*, *dmrt93B*, and *dmrt1/6*), and two ancestral surrounding genes (*kank1/4* and *elval2/4*). (2) A common ancestor of vertebrata might have possessed four *dmrt* family genes, inclduing *dmrt1/6*, *dmrt2a/2b*, *dmrt3*, and *dmrt4/5*. The four *dmrt* family genes and their conserved surrounding genes are distributed in tandem on two pairs of chromosomes, specifically *kank1/4–dmrt1/6–dmrt3–dmrt2a/2b–dmrt4/5–elalv2/4* and *kank1/4–dmrt1/6–dmrt2a/2b–dmrt4/5–elalv2/4*. (3) After three rounds (3R) of whole genome duplication, the syntenies of *kank1–dmrt1–dmrt3–dmrt2a*, *dmrt4–elavl2–caap1*, *kank4–lrp8–dmrt2b*, and *dmrt5–elavl4* are conserved in fish. (4) *dmrt6* is lost in most fish species, although some retained it with downstream *kank4–lrp8*, or it was recombined to other chromosomes with a location between *plec* and *eppk1*.

We should note that our present study has several limitations. First, although fish are the most numerous vertebrates on earth, whole genome sequences are currently available for only a small fraction of fish. In this study, 17 representative fish species from 10 superorders were selected for analysis; however, the number and coverage of species was still insufficient and may have limited the generalizability of our results. Second, this study was based on fish species with known genome sequences, which may have affected the accuracy of data analysis because genome assembly techniques and quality vary significantly among species. For example, since coelacanth genomes are not assembled to the chromosomal level, our syntenic analysis of their genes is affected. Third, it is expected that as sequencing coverage and quality increase for fish genomes, future studies will be able to confirm and expand findings and generalizability from the present study.

In this study, we applied bioinformatics methods to perform phylogenetic and synteny analyses on *dmrt* genes in 17 fish speices. (1) All the examined fish species have *dmrt1–5* and most fish species harbored two *dmrt2* paralogs (*dmrt2a* and *dmrt2b*). Phyletic evolution and structure of *dmrt1∼5* and *dmrt2b* genes were relatively conserved in most of fish. The *dmrt6* gene is lost in most fish genomes and less conservative. (2) Purifying selections on the *dmrt1*, *dmrt2(2a)*, *dmrt3*, and the *dmrt1–dmrt3–dmrt2(2a)* gene cluster were observed. (3) Fish with various sex-determination systems have the similar genomic conservation patterns of the *dmrt1–dmrt3–dmrt2(2a)* gene cluster. dmrt2b, dmrt4, and dmrt5 were also relatively conserved during the evolutionary process. The high conservation of the *dmrt1–dmrt3–dmrt2(2a)* gene cluster in various fish genomes suggests their crucial biological functions while various *dmrt* family members and sequences across fish species suggest different biological roles during evolution.

Furthermore, we hypothesized the evolutionary history of the *dmrt* genes in fish after fish-specific genome duplication(s). Moreover, here raised a series of new questions during the course of our data analysis. For example, in terms of evolutionary analysis, whether *dmrt2b* is homologous and functionally similar to a specific *dmrt* in higher animals, or does fish *dmrt6* have similar functions to mammalian counterpart. We anticipate that these gene trees will help to place current *dmrt* research in a proper phylogenomic context. Our present study will provide a solid molecular basis for functional research on the fish *dmrt* family and may in particular serve as genetic reference for in-depth phylogenomics studies.

## Data Availability Statement

The datasets presented in this study can be found in online repositories. The names of the repository/repositories and accession number(s) can be found in the article/Supplementary Material.

## Author Contributions

XY and QS conceived and designed the project and revised the manuscript. JD and JL performed the genomic investigations and wrote the manuscript. JH, CFS, YT, NY, CXS, XS, and SY participated in discussion and figure preparation. All authors read and approve the final manuscript.

## Conflict of Interest

The authors declare that the research was conducted in the absence of any commercial or financial relationships that could be construed as a potential conflict of interest. The reviewers JM and ZS declared a shared affiliation, with no collaboration, with one of the authors CFS, to the handling editor at the time of review.

## References

[B1] Altschul S. F. Gish W. Miller W. Myers E. W. Lipman D. J. (1990). Basic local alignment search tool. *J. Mol. Biol.* 215 403–410. 10.1016/S0022-2836(05)80360-2 2231712

[B2] Andersson L. S. Larhammar M. Memic F. Wootz H. Schwochow D. Rubin C. J. (2012). Mutations in DMRT3 affect locomotion in horses and spinal circuit function in mice. *Nature* 488 642–646. 10.1038/nature11399 22932389 PMC3523687

[B3] Balciuniene J. Bardwell V. J. Zarkower D. (2006). Mice mutant in the DM domain gene Dmrt4 are viable and fertile but have polyovular follicles. *Mol. Cell. Biol.* 26 8984–8991. 10.1128/MCB.00959-06 16982677 PMC1636805

[B4] Birney E. Clamp M. Durbin R. (2004). GeneWise and genomewise. *Genome Res.* 14 988–995. 10.1101/gr.1865504 15123596 PMC479130

[B5] Biscotti M. A. Adolfi M. C. Barucca M. Forconi M. Pallavicini A. Gerdol M. (2018). A comparative view on sex differentiation and gametogenesis genes in lungfish and coelacanths. *Genome Biol. Evol.* 10 1430–1444. 10.1093/gbe/evy101 29850809 PMC6007259

[B6] Braasch I. Postlethwait J. H. (2012). “Polyploidy in fish and the teleost genome duplication,” in *Polyploidy and Genome Evolution*, eds Soltis P. S. Soltis D. E. (Berlin, Heidelberg: Springer Berlin Heidelberg), 341–383. 10.1007/978-3-642-31442-1_17

[B7] Burtis K. C. Baker B. S. (1989). *Drosophila* doublesex gene controls somatic sexual differentiation by producing alternatively spliced mRNAs encoding related sex-specific polypeptides. *Cell* 56 997–1010. 10.1016/0092-8674(89)90633-8 2493994

[B8] Cao J. Cao Z. Wu T. (2007). Generation of antibodies against DMRT1 and DMRT4 of *Oreochromis aurea* and analysis of their expression profile in *Oreochromis aurea* tissues. *J. Genet. Genomics* 34 497–509. 10.1016/S1673-8527(07)60055-1 17601609

[B9] Cao M. Duan J. Cheng N. Zhong X. Wang Z. Hu W. (2012). Sexually dimorphic and ontogenetic expression of dmrt1, cyp19a1a and cyp19a1b in *Gobiocypris rarus*. *Comp. Biochem. Physiol. A Mol. Integr. Physiol.* 162 303–309. 10.1016/j.cbpa.2012.03.021 22504107

[B10] Chen N. (2004). Using RepeatMasker to identify repetitive elements in genomic sequences. *Curr. Protoc. Bioinformatics* Chapter 4 Unit4.10. 10.1002/0471250953.bi0410s05 18428725

[B11] Chen S. Zhang G. Shao C. Huang Q. Liu G. Zhang P. (2014). Whole-genome sequence of a flatfish provides insights into ZW sex chromosome evolution and adaptation to a benthic lifestyle. *Nat. Genet.* 46 253–260. 10.1038/ng.2890 24487278

[B12] Chen S. L. Ji X. S. Shao C. W. Li W. L. Yang J. F. Liang Z. (2012). Induction of mitogynogenetic diploids and identification of WW super-female using sex-specific SSR markers in half-smooth tongue sole (*Cynoglossus semilaevis*). *Mar. Biotechnol. (NY)* 14 120–128. 10.1007/s10126-011-9395-2 21735350

[B13] Cui Z. Liu Y. Wang W. Wang Q. Zhang N. Lin F. (2017). Genome editing reveals dmrt1 as an essential male sex-determining gene in Chinese tongue sole (*Cynoglossus semilaevis*). *Sci. Rep.* 7:42213. 10.1038/srep42213 28205594 PMC5311979

[B14] Darriba D. Taboada G. L. Doallo R. Posada D. (2011). ProtTest 3: fast selection of best-fit models of protein evolution. *Bioinformatics* 27 1164–1165. 10.1093/bioinformatics/btr088 21335321 PMC5215816

[B15] De Clercq S. Keruzore M. Desmaris E. Pollart C. Assimacopoulos S. Preillon J. (2018). DMRT5 together with DMRT3 directly controls hippocampus development and neocortical area map formation. *Cereb. Cortex* 28 493–509. 10.1093/cercor/bhw384 28031177 PMC6059253

[B16] Dong X. L. Chen S. L. (2013). Molecular cloning and expression analysis of Dmrt4 gene in half-smooth tongue sole (*Cynoglossus semilaevis*). *J. Fish. Sci. China* 20 499–505. 10.3724/sp.j.1118.2013.00499

[B17] Dong X. L. Chen S. L. Ji X. S. (2010). Molecular cloning and expression analysis of Dmrt3 gene in half-smooth tongue sole (*Cynoglossus semilaevis*). *J. Fish. China* 34 829–835. 10.1007/s10695-018-0472-6 29404821

[B18] El-Mogharbel N. Wakefield M. Deakin J. E. Tsend-Ayush E. Grutzner F. Alsop A. (2007). DMRT gene cluster analysis in the platypus: new insights into genomic organization and regulatory regions. *Genomics* 89 10–21. 10.1016/j.ygeno.2006.07.017 16962738

[B19] Erdman S. E. Burtis K. C. (1993). The *Drosophila* doublesex proteins share a novel zinc finger related DNA binding domain. *EMBO J.* 12 527–535. 10.1002/j.1460-2075.1993.tb05684.x 8440242 PMC413235

[B20] Fajkowska M. Rzepkowska M. Adamek D. Ostaszewska T. Szczepkowski M. (2016). Expression of dmrt1 and vtg genes during gonad formation, differentiation and early maturation in cultured Russian sturgeon *Acipenser gueldenstaedtii*. *J. Fish. Biol.* 89 1441–1449. 10.1111/jfb.12992 27239004

[B21] Fernandino J. I. Hattori R. S. Shinoda T. Kimura H. Strobl-Mazzulla P. H. Strussmann C. A. (2008). Dimorphic expression of dmrt1 and cyp19a1 (ovarian aromatase) during early gonadal development in pejerrey, *Odontesthes bonariensis*. *Sex Dev.* 2 316–324. 10.1159/000195681 19276634

[B22] Forconi M. Canapa A. Barucca M. Biscotti M. A. Capriglione T. Buonocore F. (2013). Characterization of sex determination and sex differentiation genes in Latimeria. *PLoS One* 8:e56006. 10.1371/journal.pone.0056006 23634199 PMC3636272

[B23] Frazer K. A. Pachter L. Poliakov A. Rubin E. M. Dubchak I. (2004). VISTA: computational tools for comparative genomics. *Nucleic Acids Res.* 32 W273–W279. 10.1093/nar/gkh458 15215394 PMC441596

[B24] Glennon R. Gomelsky B. Schneider K. Kelly A. Haukenes A. (2012). Evidence of female heterogamety in largemouth bass, based on sex ratio of gynogenetic progeny. *North Am. J. Aquac.* 74 537–540. 10.1080/15222055.2012.700906

[B25] Graf M. Teo Qi-Wen E. R. Sarusie M. V. Rajaei F. Winkler C. (2015). Dmrt5 controls corticotrope and gonadotrope differentiation in the zebrafish pituitary. *Mol. Endocrinol.* 29 187–199. 10.1210/me.2014-1176 25489906 PMC5414757

[B26] Gu H. T. Liu Q. Q. Situ J. X. Jiang D. N. Chen H. P. Wu T. L. (2019). Cloning and expression of Dmrt5 gene in *Scatophagus argus*. *J. Hainan Trop. Ocean Univ.* 26 9–15.

[B27] Guan G. Kobayashi T. Nagahama Y. (2000). Sexually dimorphic expression of two types of DM (Doublesex/Mab-3)-domain genes in a teleost fish, the Tilapia (*Oreochromis niloticus*). *Biochem. Biophys. Res. Commun.* 272 662–666. 10.1006/bbrc.2000.2840 10860811

[B28] Guo Y. Cheng H. Huang X. Gao S. Yu H. Zhou R. (2005). Gene structure, multiple alternative splicing, and expression in gonads of zebrafish Dmrt1. *Biochem. Biophys. Res. Commun.* 330 950–957. 10.1016/j.bbrc.2005.03.066 15809088

[B29] Guo Y. Li Q. Gao S. Zhou X. He Y. Shang X. (2004). Molecular cloning, characterization, and expression in brain and gonad of Dmrt5 of zebrafish. *Biochem. Biophys. Res. Commun.* 324 569–575. 10.1016/j.bbrc.2004.09.085 15474464

[B30] He C. L. Du J. L. Wu G. C. Lee Y. H. Sun L. T. Chang C. F. (2003). Differential Dmrt1 transcripts in gonads of the protandrous black porgy, *Acanthopagrus schlegeli*. *Cytogenet. Genome Res.* 101 309–313. 10.1159/000074354 14685000

[B31] He Z. Zhang H. Gao S. Lercher M. J. Chen W. H. Hu S. (2016). Evolview v2: an online visualization and management tool for customized and annotated phylogenetic trees. *Nucleic Acids Res.* 44 W236–W241. 10.1093/nar/gkw370 27131786 PMC4987921

[B32] Herpin A. Schartl M. (2011). Dmrt1 genes at the crossroads: a widespread and central class of sexual development factors in fish. *FEBS J.* 278 1010–1019. 10.1111/j.1742-4658.2011.08030.x 21281449

[B33] Hodgkin J. (2002). The remarkable ubiquity of DM domain factors as regulators of sexual phenotype: ancestry or aptitude? *Genes Dev.* 16 2322–2326. 10.1101/gad.1025502 12231620

[B34] Hong C. S. Park B. Y. Saint-Jeannet J. P. (2007). The function of Dmrt genes in vertebrate development: it is not just about sex. *Dev. Biol.* 310 1–9. 10.1016/j.ydbio.2007.07.035 17720152

[B35] Huang X. Guo Y. Shui Y. Gao S. Yu H. Cheng H. (2005a). Multiple alternative splicing and differential expression of dmrt1 during gonad transformation of the rice field eel. *Biol. Reprod.* 73 1017–1024. 10.1095/biolreprod.105.041871 16014815

[B36] Huang X. Hong C. S. O’Donnell M. Saint-Jeannet J. P. (2005b). The doublesex-related gene, XDmrt4, is required for neurogenesis in the olfactory system. *Proc. Natl. Acad. Sci. U.S.A.* 102 11349–11354. 10.1073/pnas.0505106102 16061812 PMC1183594

[B37] Jeng S. R. Wu G. C. Yueh W. S. Kuo S. F. Dufour S. Chang C. F. (2019). Dmrt1 (doublesex and mab-3-related transcription factor 1) expression during gonadal development and spermatogenesis in the Japanese eel. *Gen. Comp. Endocrinol.* 279 154–163. 10.1016/j.ygcen.2019.03.012 30902612

[B38] Jiang D. N. Peng Y. Q. Mustapha U. F. Gu H. T. Deng S. P. Chen H. P. (2019). molecular cloning and expression profile of Dmrt4 in spotted scat (*Scatophagus argus*). *J. Guangdong Ocean Univ.* 39 7–13.

[B39] Jiang S. Chen X. W. Shi Z. Y. Li Q. (2012). Expression of *Dmrt2* gene in *Carassius auratus*. *J. Shanghai Ocean Univ.* 21 701–708.

[B40] Johnsen H. Andersen O. (2012). Sex dimorphic expression of five dmrt genes identified in the Atlantic cod genome. The fish-specific dmrt2b diverged from dmrt2a before the fish whole-genome duplication. *Gene* 505 221–232. 10.1016/j.gene.2012.06.021 22749781

[B41] Johnsen H. Seppola M. Torgersen J. S. Delghandi M. Andersen O. (2010). Sexually dimorphic expression of dmrt1 in immature and mature Atlantic cod (*Gadus morhua* L.). *Comp. Biochem. Physiol. B Biochem. Mol. Biol.* 156 197–205. 10.1016/j.cbpb.2010.03.009 20363354

[B42] Kasahara M. Naruse K. Sasaki S. Nakatani Y. Qu W. Ahsan B. (2007). The medaka draft genome and insights into vertebrate genome evolution. *Nature* 447 714–719. 10.1038/nature05846 17554307

[B43] Katoh K. Misawa K. Kuma K. Miyata T. (2002). MAFFT: a novel method for rapid multiple sequence alignment based on fast Fourier transform. *Nucleic Acids Res.* 30 3059–3066. 10.1093/nar/gkf436 12136088 PMC135756

[B44] Kawamata M. Nishimori K. (2006). Mice deficient in Dmrt7 show infertility with spermatogenic arrest at pachytene stage. *FEBS Lett.* 580 6442–6446. 10.1016/j.febslet.2006.10.066 17098235

[B45] Kim S. Kettlewell J. R. Anderson R. C. Bardwell V. J. Zarkower D. (2003). Sexually dimorphic expression of multiple doublesex-related genes in the embryonic mouse gonad. *Gene Expr. Patterns* 3 77–82. 10.1016/s1567-133x(02)00071-6 12609607

[B46] Kobayashi T. Kajiura-Kobayashi H. Guan G. Nagahama Y. (2008). Sexual dimorphic expression of DMRT1 and Sox9a during gonadal differentiation and hormone-induced sex reversal in the teleost fish Nile tilapia (*Oreochromis niloticus*). *Dev. Dyn.* 237 297–306. 10.1002/dvdy.21409 18095345

[B47] Kobayashi T. Matsuda M. Kajiura-Kobayashi H. Suzuki A. Saito N. Nakamoto M. (2004). Two DM domain genes, DMY and DMRT1, involved in testicular differentiation and development in the medaka, *Oryzias latipes*. *Dev. Dyn.* 231 518–526. 10.1002/dvdy.20158 15376325

[B48] Kondo M. Froschauer A. Kitano A. Nanda I. Hornung U. Volff J. N. (2002). Molecular cloning and characterization of DMRT genes from the medaka *Oryzias latipes* and the platyfish *Xiphophorus maculatus*. *Gene* 295 213–222. 10.1016/s0378-1119(02)00692-3 12354656

[B49] Konno D. Iwashita M. Satoh Y. Momiyama A. Abe T. Kiyonari H. (2012). The mammalian DM domain transcription factor Dmrta2 is required for early embryonic development of the cerebral cortex. *PLoS One* 7:e46577. 10.1371/journal.pone.0046577 23056351 PMC3462758

[B50] Li L. Mao A. Wang P. Ning G. Cao Y. Wang Q. (2018). Endodermal pouch-expressed dmrt2b is important for pharyngeal cartilage formation. *Biol Open* 7:bio035444. 10.1242/bio.035444 30341107 PMC6310889

[B51] Li Q. Zhou X. Guo Y. Shang X. Chen H. Lu H. (2008). Nuclear localization, DNA binding and restricted expression in neural and germ cells of zebrafish Dmrt3. *Biol. Cell* 100 453–463. 10.1042/BC20070114 18282142

[B52] Lien S. Koop B. F. Sandve S. R. Miller J. R. Kent M. P. Nome T. (2016). The Atlantic salmon genome provides insights into rediploidization. *Nature* 533 200–205. 10.1038/nature17164 27088604 PMC8127823

[B53] Lin Q. Mei J. Li Z. Zhang X. Zhou L. Gui J. F. (2017). Distinct and cooperative roles of amh and dmrt1 in self-renewal and differentiation of male germ cells in zebrafish. *Genetics* 207 1007–1022. 10.1534/genetics.117.300274 28893856 PMC5676237

[B54] Liu S. Gui J. F. (2011). Molecular characterization and functional analysis of gibel carp dmrt2b. *Acta Hydrobiol. Sin.* 35 379–383.

[B55] Liu S. Li Z. Gui J. F. (2009). Fish-specific duplicated dmrt2b contributes to a divergent function through Hedgehog pathway and maintains left-right asymmetry establishment function. *PLoS One* 4:e7261. 10.1371/journal.pone.0007261 19789708 PMC2749440

[B56] Lourenco R. Lopes S. S. Saude L. (2010). Left-right function of dmrt2 genes is not conserved between zebrafish and mouse. *PLoS One* 5:e14438. 10.1371/journal.pone.0014438 21203428 PMC3010978

[B57] Loytynoja A. Goldman N. (2005). An algorithm for progressive multiple alignment of sequences with insertions. *Proc. Natl. Acad. Sci. U.S.A.* 102 10557–10562. 10.1073/pnas.0409137102 16000407 PMC1180752

[B58] Lu C. Wu J. Xiong S. Zhang X. Zhang J. Mei J. (2017). MicroRNA-203a regulates fast muscle differentiation by targeting dmrt2a in zebrafish embryos. *Gene* 625 49–54. 10.1016/j.gene.2017.05.012 28483596

[B59] Lyu Q. Hu J. Yang X. Liu X. Chen Y. Xiao L. (2019). Expression profiles of dmrts and foxls during gonadal development and sex reversal induced by 17alpha-methyltestosterone in the orange-spotted grouper. *Gen. Comp. Endocrinol.* 274 26–36. 10.1016/j.ygcen.2018.12.014 30594589

[B60] Ma L. Wang W. Yang X. Jiang J. Song H. Jiang H. (2014). Characterization of the Dmrt1 gene in the black rockfish *Sebastes schlegeli* revealed a remarkable sex-dimorphic expression. *Fish. Physiol. Biochem.* 40 1263–1274. 10.1007/s10695-014-9921-z 24566822

[B61] Marchand O. Govoroun M. D’Cotta H. McMeel O. Lareyre J. J. Bernot A. (2000). DMRT1 expression during gonadal differentiation and spermatogenesis in the rainbow trout, *Oncorhynchus mykiss*. *Biochim. Biophys. Acta* 1493 180–187. 10.1016/s0167-4781(00)00186-x 10978520

[B62] Matson C. K. Murphy M. W. Sarver A. L. Griswold M. D. Bardwell V. J. Zarkower D. (2011). DMRT1 prevents female reprogramming in the postnatal mammalian testis. *Nature* 476 101–104. 10.1038/nature10239 21775990 PMC3150961

[B63] Matson C. K. Zarkower D. (2012). Sex and the singular DM domain: insights into sexual regulation, evolution and plasticity. *Nat. Rev. Genet.* 13 163–174. 10.1038/nrg3161 22310892 PMC3595575

[B64] Mawaribuchi S. Ito Y. Ito M. (2019). Independent evolution for sex determination and differentiation in the DMRT family in animals. *Biol. Open* 8:bio041962. 10.1242/bio.041962 31399444 PMC6737965

[B65] Mei J. Gui J. F. (2015). Genetic basis and biotechnological manipulation of sexual dimorphism and sex determination in fish. *Sci. China Life Sci.* 58 124–136. 10.1007/s11427-014-4797-9 25563981

[B66] Meng A. Moore B. Tang H. Yuan B. Lin S. (1999). A *Drosophila* doublesex-related gene, terra, is involved in somitogenesis in vertebrates. *Development* 126 1259–1268. 10021344 10.1242/dev.126.6.1259

[B67] Miyake Y. Sakai Y. Kuniyoshi H. (2012). Molecular cloning and expression profile of sex-specific genes, Figla and Dmrt1, in the protogynous hermaphroditic fish, *Halichoeres poecilopterus*. *Zoolog. Sci.* 29 690–701. 10.2108/zsj.29.690 23030342

[B68] Moniot B. Berta P. Scherer G. Sudbeck P. Poulat F. (2000). Male specific expression suggests role of DMRT1 in human sex determination. *Mech. Dev.* 91 323–325. 10.1016/s0925-4773(99)00267-1 10704857

[B69] Muralidharan B. Keruzore M. Pradhan S. J. Roy B. Shetty A. S. Kinare V. (2017). Dmrt5, a novel neurogenic factor, reciprocally regulates Lhx2 to control the neuron-glia cell-fate switch in the developing hippocampus. *J. Neurosci.* 37 11245–11254. 10.1523/JNEUROSCI.1535-17.2017 29025924 PMC5688529

[B70] Nelson J. S. Grande T. C. Wilson M. V. H. (2016). *Fishes of the World.* Hoboken, NJ: Wiley.

[B71] Nomura K. Nakajima J.-I. Ohta H. Kagawa H. Tanaka H. Unuma T. (2004). Induction of triploidy by heat shock in the Japanese eel *Anguilla japonica*. *Fish. Sci.* 70 247–255. 10.1111/j.1444-2906.2003.00798.x

[B72] Otake H. Shinomiya A. Matsuda M. Hamaguchi S. Sakaizumi M. (2006). Wild-derived XY sex-reversal mutants in the medaka, *Oryzias latipes*. *Genetics* 173 2083–2090. 10.1534/genetics.106.058941 16702419 PMC1569717

[B73] Ottolenghi C. Fellous M. Barbieri M. McElreavey K. (2002). Novel paralogy relations among human chromosomes support a link between the phylogeny of doublesex-related genes and the evolution of sex determination. *Genomics* 79 333–343. 10.1006/geno.2002.6711 11863363

[B74] Panara V. Budd G. E. Janssen R. (2019). Phylogenetic analysis and embryonic expression of panarthropod Dmrt genes. *Front. Zool.* 16:23. 10.1186/s12983-019-0322-0 31303887 PMC6604209

[B75] Portela-Bens S. Merlo M. A. Rodriguez M. E. Cross I. Manchado M. Kosyakova N. (2017). Integrated gene mapping and synteny studies give insights into the evolution of a sex proto-chromosome in *Solea senegalensis*. *Chromosoma* 126 261–277. 10.1007/s00412-016-0589-2 27080536

[B76] Raymond C. S. Shamu C. E. Shen M. M. Seifert K. J. Hirsch B. Hodgkin J. (1998). Evidence for evolutionary conservation of sex-determining genes. *Nature* 391 691–695. 10.1038/35618 9490411

[B77] Robledo D. Ribas L. Cal R. Sanchez L. Piferrer F. Martinez P. (2015). Gene expression analysis at the onset of sex differentiation in turbot (*Scophthalmus maximus*). *BMC Genomics* 16:973. 10.1186/s12864-015-2142-8 26581195 PMC4652359

[B78] Ronquist F. Teslenko M. van der Mark P. Ayres D. L. Darling A. Hohna S. (2012). MrBayes 3.2: efficient Bayesian phylogenetic inference and model choice across a large model space. *Syst. Biol.* 61 539–542. 10.1093/sysbio/sys029 22357727 PMC3329765

[B79] Sahoo L. Sahoo S. Mohanty M. Sankar M. Dixit S. Das P. (2019). Molecular characterization, computational analysis and expression profiling of Dmrt1 gene in Indian major carp, *Labeo rohita* (Hamilton 1822). *Anim. Biotechnol.* 1–14. 10.1080/10495398.2019.1707683 31880491

[B80] Saude L. Lourenco R. Goncalves A. Palmeirim I. (2005). terra is a left-right asymmetry gene required for left-right synchronization of the segmentation clock. *Nat. Cell. Biol.* 7 918–920. 10.1038/ncb1294 16136187

[B81] Schultheis C. Bohne A. Schartl M. Volff J. N. Galiana-Arnoux D. (2009). Sex determination diversity and sex chromosome evolution in poeciliid fish. *Sex. Dev.* 3 68–77. 10.1159/000223072 19684452

[B82] Seo K. W. Wang Y. Kokubo H. Kettlewell J. R. Zarkower D. A. Johnson R. L. (2006). Targeted disruption of the DM domain containing transcription factor Dmrt2 reveals an essential role in somite patterning. *Dev. Biol.* 290 200–210. 10.1016/j.ydbio.2005.11.027 16387292

[B83] Sheng Y. Chen B. Zhang L. Luo M. Cheng H. Zhou R. (2014). Identification of Dmrt genes and their up-regulation during gonad transformation in the swamp eel (*Monopterus albus*). *Mol. Biol. Rep.* 41 1237–1245. 10.1007/s11033-013-2968-6 24390316

[B84] Shirak A. Seroussi E. Cnaani A. Howe A. E. Domokhovsky R. Zilberman N. (2006). Amh and Dmrta2 genes map to tilapia (*Oreochromis* spp.) linkage group 23 within quantitative trait locus regions for sex determination. *Genetics* 174 1573–1581. 10.1534/genetics.106.059030 16951079 PMC1667067

[B85] Slater G. S. Birney E. (2005). Automated generation of heuristics for biological sequence comparison. *BMC Bioinformatics* 6:31. 10.1186/1471-2105-6-31 15713233 PMC553969

[B86] Smith C. A. Hurley T. M. McClive P. J. Sinclair A. H. (2002). Restricted expression of DMRT3 in chicken and mouse embryos. *Mech. Dev.* 119(Suppl. 1) S73–S76. 10.1016/s0925-4773(03)00094-7 14516663

[B87] Smith E. K. Guzman J. M. Luckenbach J. A. (2013). Molecular cloning, characterization, and sexually dimorphic expression of five major sex differentiation-related genes in a Scorpaeniform fish, sablefish (*Anoplopoma fimbria*). *Comp. Biochem. Physiol. B Biochem. Mol. Biol.* 165 125–137. 10.1016/j.cbpb.2013.03.011 23507626

[B88] Su L. Zhou F. Ding Z. Gao Z. Wen J. Wei W. (2015). Transcriptional variants of Dmrt1 and expression of four Dmrt genes in the blunt snout bream, *Megalobrama amblycephala*. *Gene* 573 205–215. 10.1016/j.gene.2015.07.044 26188158

[B89] Su L. N. Ding Z. J. Li H. Zhou F. J. Wang W. M. Liu H. (2013). Molecular cloning and expression analysis of Dmrt4 gene in *Megalobrama amblycephala*. *J. Huazhong Agric. Univ.* 32 110–116.

[B90] Sun C. Li J. Dong J. Niu Y. Hu J. Lian J. (2020). Chromosome-level genome assembly for the largemouth bass *Micropterus salmoides* provides insights into adaptation to fresh and brackish water. *Mol. Ecol. Resour.* 10.1111/1755-0998.13256 [Epub ahead of print]. 32985096

[B91] Tsend-Ayush E. Lim S. L. Pask A. J. Hamdan D. D. Renfree M. B. Grutzner F. (2009). Characterisation of ATRX, DMRT1, DMRT7 and WT1 in the platypus (*Ornithorhynchus anatinus*). *Reprod. Fertil. Dev.* 21 985–991. 10.1071/RD09090 19874722

[B92] Vandeputte M. Dupont-Nivet M. Chavanne H. Chatain B. (2007). A polygenic hypothesis for sex determination in the European sea bass *Dicentrarchus labrax*. *Genetics* 176 1049–1057. 10.1534/genetics.107.072140 17435246 PMC1894574

[B93] Veith A. M. Klattig J. Dettai A. Schmidt C. Englert C. Volff J. N. (2006a). Male-biased expression of X-chromosomal DM domain-less Dmrt8 genes in the mouse. *Genomics* 88 185–195. 10.1016/j.ygeno.2006.01.003 16488114

[B94] Veith A. M. Schafer M. Kluver N. Schmidt C. Schultheis C. Schartl M. (2006b). Tissue-specific expression of dmrt genes in embryos and adults of the platyfish *Xiphophorus maculatus*. *Zebrafish* 3 325–337. 10.1089/zeb.2006.3.325 18377213

[B95] Volff J. N. Schartl M. (2001). Variability of genetic sex determination in poeciliid fishes. *Genetica* 111 101–110. 10.1023/a:1013795415808 11841158

[B96] Volff J. N. Zarkower D. Bardwell V. J. Schartl M. (2003). Evolutionary dynamics of the DM domain gene family in metazoans. *J. Mol. Evol.* 57(Suppl. 1) S241–S249. 10.1007/s00239-003-0033-0 15008421

[B97] Wang D. Zhang S. He F. Zhu J. Hu S. Yu J. (2009). How do variable substitution rates influence Ka and Ks calculations? *Genomics Proteomics Bioinformatics* 7 116–127. 10.1016/S1672-0229(08)60040-6 19944384 PMC5054415

[B98] Wang D. P. Wan H. L. Zhang S. Yu J. (2009). Gamma-MYN: a new algorithm for estimating Ka and Ks with consideration of variable substitution rates. *Biol. Direct.* 4:20. 10.1186/1745-6150-4-20 19531225 PMC2702329

[B99] Wang H. (2013). The bioinformatical analysis on DMRT family and possible role of Dmrt4/Dmrt6 in the development of tilapia gonad. *Southwest Univ.*

[B100] Wang H. Piferrer F. Chen S. Shen Z. (2018). “Epigenetics of sex determination and differentiation in fish,” in *Sex Control in Aquaculture*, eds Wang H. P. Piferrer F. Chen S. L. Shen Z. G. (Hoboken, NJ: John Wiley & Sons), 65–83. 10.1002/9781119127291.ch3

[B101] Wang J. H. Miao L. Li M. Y. Guo X. F. Pan N. Chen Y. Y. (2014). Cloning the Dmrt1 and DmrtA2 genes of ayu (*Plecoglossus altivelis*) and mapping their expression in adult, larval, and embryonic stages. *Zool. Res.* 35 99–107. 10.11813/j.issn.0254-5853.2014.2.099 PMC5042934 24668652

[B102] Webster K. A. Schach U. Ordaz A. Steinfeld J. S. Draper B. W. Siegfried K. R. (2017). Dmrt1 is necessary for male sexual development in zebrafish. *Dev. Biol.* 422 33–46. 10.1016/j.ydbio.2016.12.008 27940159 PMC5777149

[B103] Wei L. Li X. Li M. Tang Y. Wei J. Wang D. (2019). Dmrt1 directly regulates the transcription of the testis-biased Sox9b gene in Nile tilapia (*Oreochromis niloticus*). *Gene* 687 109–115. 10.1016/j.gene.2018.11.016 30415011

[B104] Wen A. You F. Tan X. Sun P. Ni J. Zhang Y. (2009). Expression pattern of dmrt4 from olive flounder (*Paralichthys olivaceus*) in adult gonads and during embryogenesis. *Fish. Physiol. Biochem.* 35 421–433. 10.1007/s10695-008-9267-5 18841490

[B105] Wexler J. R. Plachetzki D. C. Kopp A. (2014). Pan-metazoan phylogeny of the DMRT gene family: a framework for functional studies. *Dev. Genes Evol.* 224 175–181. 10.1007/s00427-014-0473-0 24903586

[B106] Winkler C. Hornung U. Kondo M. Neuner C. Duschl J. Shima A. (2004). Developmentally regulated and non-sex-specific expression of autosomal dmrt genes in embryos of the Medaka fish (*Oryzias latipes*). *Mech. Dev.* 121 997–1005. 10.1016/j.mod.2004.03.018 15210205

[B107] Wu G. C. Chang C. F. (2018). Primary males guide the femaleness through the regulation of testicular Dmrt1 and ovarian Cyp19a1a in protandrous black porgy. *Gen. Comp. Endocrinol.* 261 198–202. 10.1016/j.ygcen.2017.01.033 28188743

[B108] Xu S. Xia W. Zohar Y. Gui J. F. (2013). Zebrafish dmrta2 regulates the expression of cdkn2c in spermatogenesis in the adult testis. *Biol. Reprod.* 88:14. 10.1095/biolreprod.112.105130 23175770

[B109] Yamaguchi A. Lee K. H. Fujimoto H. Kadomura K. Yasumoto S. Matsuyama M. (2006). Expression of the DMRT gene and its roles in early gonadal development of the Japanese pufferfish *Takifugu rubripes*. *Comp. Biochem. Physiol. Part D Genomics Proteomics* 1 59–68. 10.1016/j.cbd.2005.08.003 20483235

[B110] Yan N. Hu J. Li J. Dong J. Sun C. Ye X. (2019). Genomic organization and sexually dimorphic expression of the Dmrt1 gene in largemouth bass (*Micropterus salmoides*). *Comp. Biochem. Physiol. B Biochem. Mol. Biol.* 234 68–77. 10.1016/j.cbpb.2019.05.005 31078703

[B111] Yang Z. Nielsen R. (2000). Estimating synonymous and nonsynonymous substitution rates under realistic evolutionary models. *Mol. Biol. Evol.* 17 32–43. 10.1093/oxfordjournals.molbev.a026236 10666704

[B112] Yoshimoto S. Ikeda N. Izutsu Y. Shiba T. Takamatsu N. Ito M. (2010). Opposite roles of DMRT1 and its W-linked paralogue, DM-W, in sexual dimorphism of *Xenopus laevis*: implications of a ZZ/ZW-type sex-determining system. *Development* 137 2519–2526. 10.1242/dev.048751 20573695

[B113] Yoshizawa A. Nakahara Y. Izawa T. Ishitani T. Tsutsumi M. Kuroiwa A. (2011). Zebrafish Dmrta2 regulates neurogenesis in the telencephalon. *Genes Cells* 16 1097–1109. 10.1111/j.1365-2443.2011.01555.x 22023386

[B114] Zarkower D. (2001). Establishing sexual dimorphism: conservation amidst diversity? *Nat. Rev. Genet.* 2 175–185. 10.1038/35056032 11256069

[B115] Zhang T. Murphy M. W. Gearhart M. D. Bardwell V. J. Zarkower D. (2014). The mammalian Doublesex homolog DMRT6 coordinates the transition between mitotic and meiotic developmental programs during spermatogenesis. *Development* 141 3662–3671. 10.1242/dev.113936 25249458 PMC4197572

[B116] Zhang X. Wang H. Li M. Cheng Y. Jiang D. Sun L. (2014). Isolation of doublesex- and mab-3-related transcription factor 6 and its involvement in spermatogenesis in tilapia. *Biol. Reprod.* 91:136. 10.1095/biolreprod.114.121418 25320148

[B117] Zhang Z. Li J. Yu J. (2006). Computing Ka and Ks with a consideration of unequal transitional substitutions. *BMC Evol. Biol.* 6:44. 10.1186/1471-2148-6-44 16740169 PMC1552089

[B118] Zhou X. Li Q. Lu H. Chen H. Guo Y. Cheng H. (2008). Fish specific duplication of Dmrt2: characterization of zebrafish Dmrt2b. *Biochimie* 90 878–887. 10.1016/j.biochi.2008.02.021 18358846

[B119] Zhu L. Wilken J. Phillips N. B. Narendra U. Chan G. Stratton S. M. (2000). Sexual dimorphism in diverse metazoans is regulated by a novel class of intertwined zinc fingers. *Genes Dev.* 14 1750–1764. 10898790 PMC316782

[B120] Zhu Y. Cui Z. Yang Y. Xu W. Shao C. Fu X. (2019). Expression analysis and characterization of dmrt2 in Chinese tongue sole (*Cynoglossus semilaevis*). *Theriogenology* 138 1–8. 10.1016/j.theriogenology.2019.06.035 31279050

