# Supplementary material for: Comparative Genomics Studies on the dmrt Gene Family in Fish
Source: Front Genet. 2020 Nov 12;11:563947. doi: 10.3389/fgene.2020.563947 (PMC7689362; doi:10.3389/fgene.2020.563947)
Supplement: Supplementary file 1 [file Table_1.DOCX]

**Table S1.** Information of the vertebrate species used in this study.

| **Class** | **Subdivision** | **Species Name** | **Common Name** | **Taxon ID** | **Ensembl Assembly** | **Accession** |
| --- | --- | --- | --- | --- | --- | --- |
| **Mammals** | *-* | *H. sapiens* | Human | 9606 | GRCh38.p12 | GCA_000001405.27 |
|  | *-* | *M. musculus* | Mouse | 10090 | GRCm38.p6 | GCA_00001635.8 |
| **Aves** | *-* | *G. gallus* | Chicken | 9031 | GRCg6a | GCA_000002315.5 |
|  | *-* | *T. guttata* | Zebra finch | 59729 | taeGut3.2.4 | GCA_003957565.2 |
| **Reptile** | *-* | *A. carolinensis* | Anole lizard | 28377 | AnoCar2.0 | GCA_000090745.1 |
| **Amphibian** | *-* | *X. tropicalis* | Clawed frog | 8364 | JGI 4.2 | GCA_00004195.1 |
| **Fishes (Actinopterygii)** | **Percomorpha** | *M. salmoides* | Largemouth bass | 27706 | - | Unpublished |
|  |  | *L. calcarifer* | Asian seabass | 8187 | ASB_HGAPassembly_v1 | GCA_001640805.1 |
|  |  | *D. labrax* | European seabass | 13489 | - | GCA_000689215.1 |
|  |  | *T. rubripes* | Japanese pufferfish | 31033 | FUGU5 | GCA_000180615.2 |
|  |  | *C. semilaevis* | Tongue sole | 244447 | Cse_v1.0 | GCA_000523025.1 |
|  |  | *G. aculeatus* | Stickleback | 69293 | BROAD S1 | VDFJ00000000.1 |
|  | **Atherinomorpha** | *O. latipes* | Japanese medaka | 8090 | ASM223467v1 | GCA_002234675.1 |
|  |  | *X. maculatus* | Southern platyﬁsh | 8083 | X_maculatus-5.0-male | GCA_002775205.2 |
|  | **Protacanthopterygii** | *S. salar* | Atlantic salmon | 8030 | - | GCA_000233375.4 |
|  | **Paracanthopterygii** | *G. morhua* | Atlantic cod | 8049 | gadMor1 | - |
|  | **Ostariophysi** | *I. punetaus* | Channel catfish | 7998 | IpCoco_1.2 | GCA_001660625.1 |
|  |  | *E. electricus* | Electronic eel | 8005 | Ee_SOAP_WITH_SSPACE | GCA_003665695.2 |
|  | **Clupeomorpha** | *C. harengus* | Atlantic herring | 7950 | Ch_v2.0.2*803 | GCA_000966335.1 |
|  | **Elopomorpha** | *A. japonica* | Japanese eel | 7937 | - | GCA_003597225.1 |
|  | **Osteoglossomorpha** | *S. formosus* | Asian arowana | 113540 | ASM162426v1 | GCA_001624265.1 |
|  | **Holostei** | *L. oculatus* | Spotted gar | 7918 | LepOcu1 | GCA_000242695.1 |
| **Fishes (Coelacanthiformes)** | **Coelacanthiformes** | *L. chalumnae* | Coelacanth | 7897 | LatCha1 | GCA_000225785.1 |

* The genome sequence of largemouth bass is not publicly accessible.
