# Supplementary material for: Comparative Genomics Studies on the dmrt Gene Family in Fish
Source: Front Genet. 2020 Nov 12;11:563947. doi: 10.3389/fgene.2020.563947 (PMC7689362; doi:10.3389/fgene.2020.563947)
Supplement: Supplementary file 2 [file Table_2.DOCX]

**Table S2.** Accession numbers of the known *dmrt* sequences.

| **Class** | **Subdivision** | **Species** | **Common Name** | ***DMRTs*** | **Accession Number** | | **Source** |
| --- | --- | --- | --- | --- | --- | --- | --- |
|  |  |  |  |  | **Nucleotide Sequence** | **Protein Sequence** | |
| **Mammals** | *-* | *H. sapiens* | Human | *1* | NM_021951.3 | NP_068770.2 | NCBI |
|  |  |  |  | *2* | NM_181872.4 | NP_870987.2 | NCBI |
|  |  |  |  | *3* | NM_021240.4 | NP_067063.1 | NCBI |
|  |  |  |  | *4* | NM_022160.3 | NP_071443.2 | NCBI |
|  |  |  |  | *5* | NM_032110.3 | NP_115486.1 | NCBI |
|  |  |  |  | *6* | NM_033067.2 | NP_149056.1 | NCBI |
|  |  |  |  | *7* | NM_001040283.3 | NP_001035373.1 | NCBI |
|  |  |  |  | *8* | NM_001080851.2 | NP_001074320.1 | NCBI |
|  | *-* | *M. musculus* | Mouse | *1* | NM_015826.5 | NP_056641.2 | NCBI |
|  |  |  |  | *2* | NM_145831.3 | NP_665830.1 | NCBI |
|  |  |  |  | *3* | NM_177360.3 | NP_796334.2 | NCBI |
|  |  |  |  | *4* | NM_175647.3 | NP_783578.1 | NCBI |
|  |  |  |  | *5* | NM_172296.2 | NP_758500.2 | NCBI |
|  |  |  |  | *6* | NM_019872.2 | NP_063925.1 | NCBI |
|  |  |  |  | *7* | NM_027732.2 | NP_082008.1 | NCBI |
|  |  |  |  | *8* | NM_001310614.1 | NP_001297543.1 | NCBI |
| **Aves** | *-* | *G. gallus* | Chicken | *1* | ENSGALG00000010160 | ENSGALP00000037648 | Ensembl |
|  |  |  |  | *2* | ENSGALG00000026790 | ENSGALP00000043296 | Ensembl |
|  |  |  |  | *3* | ENSGALG00000010161 | ENSGALP00000016504 | Ensembl |
|  |  |  |  | *5* | XM_015291226.2 | XP_015146712.1 | NCBI |
|  |  |  |  | *6* | NM_001245981.1 | NP_001232910.1 | NCBI |
|  | *-* | *T. guttata* | Zebra finch | *1* | XM_002194543.1 | XP_002194579.1 | NCBI |
|  |  |  |  | *2* | XM_030258526.1 | XP_030114386.1 | NCBI |
|  |  |  |  | *3* | XM_030258422.1 | XP_030114282.1 | NCBI |
|  |  |  |  | *5* | XM_030279016.1 | XP_030134876.1 | NCBI |
|  |  |  |  | *6* | XM_030279056.1 | XP_030134916.1 | NCBI |
| **Reptile** | *-*  *-* | *A. carolinensis*  *A. carolinensis* | Anole lizard  Anole lizard | *1* | XM_003216553.3 | XP_003216601.2 | NCBI |
|  |  |  |  | *2* | XM_008103322.2 | XP_008101529.1 | NCBI |
|  |  |  |  | *3* | XM_003216487.2 | XP_003216535.1 | NCBI |
|  |  |  |  | *4* | XM_008103476.2 | XP_008101683.1 | NCBI |
|  |  |  |  | *5* | XM_008109599.2 | XP_008107806.1 | NCBI |
|  |  |  |  | *6* | XM_016992995.1 | XP_016848484.1 | NCBI |
| **Amphibian** | *-* | *X. tropicalis* | Clawed frog | *1* | XM_018089347.1 | XP_017944836.1 | NCBI |
|  |  |  |  | *2* | ENSXETG00000033983 | ENSXETP00000060468 | Ensembl |
|  |  |  |  | *3* | NM_001256220.2 | NP_001243149.1 | Ensembl |
|  |  |  |  | *4* | ENSXETG00000005462 | ENSXETP00000012020 | Ensembl |
|  |  |  |  | *5* | ENSXETG00000002846 | ENSXETP00000006216 | Ensembl |
|  |  |  |  | *6* | XM_002931427.4 | XP_002931473.1 | NCBI |
| **Actinopterygii**  **Actinopterygii**  **Actinopterygii** | **Percomorpha** | *L. calcar* | Asian seabass | *1* | XM_018668978.1 | XP_018524494.1 | NCBI |
|  |  |  |  | *2a*  *2b* | XM_018668306.1  XM_018693994.1 | XP_018523822.1  XP_018549510.1 | NCBI |
|  |  |  |  | *3* | XM_018668307.1 | XP_018523823.1 | NCBI |
|  |  |  |  | *4* | XM_018687971.1 | XP_018543487.1 | NCBI |
|  |  |  |  | *5* | XM_018673346.1 | XP_018528862.1 | NCBI |
|  |  |  |  | *6* | XM_018698829.1 | XP_018554345.1 | NCBI |
|  |  | *T. rubripes* | Japanese pufferfish | *1* | ENSTRUG00000015699 | ENSTRUP00000040124 | Ensembl |
|  |  |  |  | *2a*  *2b* | ENSTRUG00000015666  ENSTRUG00000018484 | ENSTRUP00000040043  ENSTRUP00000047319 | Ensembl |
|  |  |  |  | *3* | ENSTRUG00000015692 | ENSTRUP00000040103 | Ensembl |
|  |  |  |  | *4* | ENSTRUG00000000035 | ENSTRUP00000000080 | Ensembl |
|  |  |  |  | *5* | ENSTRUG00000001824 | ENSTRUP00000004208 | Ensembl |
|  |  | *C. semilaevis* | Tongue sole | *1* | NM_001294232.1 | NP_001281161.1 | NCBI |
|  |  |  |  | *2a*  *2b* | ENSCSEG00000019331  ENSCSEG00000018774 | ENSCSEP00000030176  ENSCSEP00000029314 | Ensembl |
|  |  |  |  | *3* | ENSCSEG00000019346 | ENSCSEP00000030200 | Ensembl |
|  |  |  |  | *4* | ENSCSEG00000002789 | ENSCSEP00000004289 | Ensembl |
|  |  |  |  | *5* | ENSCSEG00000020108 | ENSCSEP00000031399 | Ensembl |
|  |  | *G. aculeatus* | Stickleback | *1* | ENSGACG00000014514 | ENSGACP00000019148 | Ensembl |
|  |  |  |  | *2a*  *2b* | ENSGACG00000014508  ENSGACG00000006816 | ENSGACP00000019139  ENSGACP00000009024 | Ensembl |
|  |  |  |  | *3* | ENSGACG00000014511 | ENSGACP00000019142 | Ensembl |
|  |  |  |  | *4* | ENSGACG00000019482 | ENSGACP00000025750 | Ensembl |
|  |  |  |  | *5* | ENSGACG00000011145 | ENSGACP00000014740 | Ensembl |
|  | **Atherinomorpha** | *O. latipes* | Japanese medaka | *1* | NM_001104680.2 | NP_001098150.2 | NCBI |
|  |  |  |  | *2a*  *2b* | ENSORLG00000022893  ENSORLG00000025087 | ENSORLP00000027396  ENSORLP00000029082 | NCBI |
|  |  |  |  | *3* | XM_023958132.1 | XP_023813900.1 | NCBI |
|  |  |  |  | *4* | XM_004079663.4 | XP_004079711.1 | NCBI |
|  |  |  |  | *5* | XM_023954578.1 | XP_023810346.1 | NCBI |
|  |  | *X. maculatus* | Southern platyﬁsh | *1* | ENSXMAG00000015591 | ENSXMAP00000015624 | Ensembl |
|  |  |  |  | *2a*  *2b* | ENSXMAG00000015603  ENSXMAG00000022040 | ENSXMAP00000015635  ENSXMAP00000035377 | Ensembl |
|  |  |  |  | *3* | ENSXMAG00000015600 | ENSXMAP00000015627 | Ensembl |
|  |  |  |  | *4* | ENSXMAG00000012522 | ENSXMAP00000012534 | Ensembl |
|  |  |  |  | *5* | ENSXMAG00000003327 | ENSXMAP00000003337 | Ensembl |
|  | **Protacanthopterygii** | *S. salar* | Atlantic salmon | *1* | XM_014172771.1 | XP_014028246.1 | NCBI |
|  |  |  |  | *2a1*  *2a2*  *2b1*  *2b2* | XM_014143592.1  XM_014172769.1  NM_001139597.1  XM_014123496.1 | XP_013999067.1  XP_014028244.1  NP_001133069.1  XP_013978971.1 | NCBI |
|  |  |  |  | *3a*  *3b* | XM_014143609.1  XM_014172770.1 | XP_013999084.1  XP_014028245.1 | NCBI |
|  |  |  |  | *4* | XM_014206453.1 | XP_014061928.1 | NCBI |
|  |  |  |  | *5a*  *5b* | XM_014216633.1  XM_014169489.1 | XP_014072108.1  XP_014024964.1 | NCBI |
|  | **Paracanthopterygii** | *G. morhua* | Atlantic cod | *1* | EU561663.1 | ACB97630.1 | NCBI |
|  |  |  |  | *2* | JN802284.1 | AFA46801.1 | NCBI |
|  |  |  |  | *3* | XM_030359568.1 | XP_030215428.1 | NCBI |
|  |  |  |  | *4* | XM_030338160.1 | XP_030194020.1 | NCBI |
|  |  |  |  | *5* | XM_030371676.1 | XP_030227536.1 | NCBI |
|  | **Ostariophysi**  **Ostariophysi** | *I. punctatus* | Channel catfish | *1* | XM_017452552.1 | XP_017308041.1 | NCBI |
|  |  |  |  | *2a*  *2b* | XM_017452569.1  XM_017479124.1 | XP_017308058.1  XP_017334613.1 | NCBI |
|  |  |  |  | *3* | XM_017452551.1 | XP_017308040.1 | NCBI |
|  |  |  |  | *4* | XM_017460796.1 | XP_017316285.1 | NCBI |
|  |  |  |  | *5* | XM_017468365.1 | XP_017323854.1 | NCBI |
|  |  |  |  | *6* | XM_017473141.1 | XP_017328630.1 | NCBI |
|  |  | *E. electricus* | Electric eel | *1* | XM_027029082.1 | XP_026884883.1 | NCBI |
|  |  |  |  | *2a*  *2b* | ENSEEEG00000003032  ENSEEEG00000007592 | ENSEEEP00000005780  ENSEEEP00000015270 | Ensembl |
|  |  |  |  | *3* | XM_027029072.1 | XP_026884873.1 | NCBI |
|  |  |  |  | *4* | XM_027023645.1 | XP_026879446.1 | NCBI |
|  |  |  |  | *5* | XM_027001404.1 | XP_026857205.1 | NCBI |
|  |  | *Danio rerio* | zebrafish | *1* | ENSDARG00000007349 | ENSDARP00000044360 | Ensembl |
|  |  |  |  | *2a*  *2b* | ENSDARG00000015072  ENSDARG00000070013 | ENSDARP00000155386  ENSDARP00000093167 | Ensembl |
|  |  |  |  | *3* | ENSDARG00000035290 | ENSDARP00000051145 | Ensembl |
|  |  |  |  | *5* | ENSDARG00000039412 | ENSDARP00000057589 | Ensembl |
|  | **Clupeomorpha** | *C. harengus* | Atlantic herring | *1* | XM_012828604.1 | XP_012684058.1 | NCBI |
|  |  |  |  | *2a*  *2b* | XM_012828568.1  XM_012828339.1 | XP_012684022.1  XP_012683793.1 | NCBI |
|  |  |  |  | *3* | XM_012828602.1 | XP_012684056.1 | NCBI |
|  |  |  |  | *4* | XM_012830816.1 | XP_012686270.1 | NCBI |
|  |  |  |  | *5* | XM_012821698.1 | XP_012677152.1 | NCBI |
|  | **Osteoglossomorpha** | *S. formosus* | Asian arowana | *1* | ENSSFOG00015019588 | ENSSFOP00015030528 | Ensembl |
|  |  |  |  | *2a*  *2b* | ENSSFOG00015014922  ENSSFOG00015019541 | ENSSFOP00015023208  ENSSFOP00015030490 | NCBI |
|  |  |  |  | *3* | XM_029259610.1 | XP_029115443.1 | NCBI |
|  |  |  |  | *4* | XM_029252387.1 | XP_029108220.1 | NCBI |
|  |  |  |  | *5* | XM_029254948.1 | XP_029110781.1 | NCBI |
|  | **Holostei** | *L. oculatus* | Spotted gar | *1* | ENSGACG00000014514 | ENSLOCP00000014096 | Ensembl |
|  |  |  |  | *2a*  *2b* | ENSLOCG00000011487  ENSLOCG00000006480 | ENSLOCP00000014117  ENSLOCP00000007824 | Ensembl |
|  |  |  |  | *3* | ENSLOCG00000011480 | ENSLOCP00000014107 | Ensembl |
|  |  |  |  | *4* | ENSLOCG00000012567 | ENSLOCP00000015471 | Ensembl |
|  |  |  |  | *5* | ENSLOCG00000009379 | ENSLOCP00000011440 | Ensembl |
|  |  |  |  | *6* | ENSLOCG00000006493 | ENSLOCP00000007844 | Ensembl |
| **Coelacanthiformes** | **Coelacanthiformes** | *L. chalumnae* | Coelacanth | *1* | ENSLACG00000013139 | ENSLACP00000014930 | Ensembl |
|  |  |  |  | *2* | ENSLACG00000011510 | ENSLACP00000013067 | Ensembl |
|  |  |  |  | *3* | XM_006001196.1 | XP_006001258.1 | NCBI |
|  |  |  |  | *4* | ENSLACG00000009724 | ENSLACP00000011049 | Ensembl |
|  |  |  |  | *5* | ENSLACG00000012046 | ENSLACP00000013688 | Ensembl |
|  |  |  |  | *6* | ENSLACG00000003331 | ENSLACP00000003739 | Ensembl |
|  |  | *L. menadoensis* | Indonesian coelacanth | *6* | HF562309.1 | CCP19130.1 | NCBI |
|  |  | *P. annectens* | African lungfish | *6* | MH329966.1 | AWT24633.1 | NCBI |
| **Anthozoa** | **Actiniaria** | *N. vectensis* | Sea anemone | *A* | JX559763.1 | AFV47365.1 | NCBI |
