# Supplementary material for: Comparative Genomics Studies on the dmrt Gene Family in Fish
Source: Front Genet. 2020 Nov 12;11:563947. doi: 10.3389/fgene.2020.563947 (PMC7689362; doi:10.3389/fgene.2020.563947)
Supplement: Supplementary file 3 [file Table_3.DOCX]

**Table S3.** Full names and accession numbers of genes used for the syntenic analysis.

| **Abbreviation** | **Full name** | **Accession Number** | |  |
| --- | --- | --- | --- | --- |
|  |  | **Nucleotide Sequence** | **Protein Sequence** | |
| *gas1a* | *growth arrest-specific 1a* | ENSDARG00000077298 | ENSDARP00000143663 | |
| *dapk1* | *death-associated protein kinase 1* | ENSDARG00000060093 | ENSDARP00000105928 | |
| *ctsla* | *cathepsin La* | ENSDARG00000007836 | ENSDARP00000106602 | |
| *fbp2* | *fructose-1,6-bisphosphatase 2* | ENSDARG00000012366 | ENSDARP00000019407 | |
| *fbp1a* | *fructose-1,6-bisphosphatase 1a* | ENSDARG00000021366 | ENSDARP00000004866 | |
| *kank1a* | *KN motif and ankyrin repeat domains 1a* | ENSDARG00000060102 | ENSDARP00000121899 | |
| *smarca2* | *SWI/SNF related, matrix associated, actin dependent regulator of chromatin, subfamily a, member 2* | ENSDARG00000008904 | ENSDARP00000133519 | |
| *adamts3* | *ADAM metallopeptidase with thrombospondin type 1 motif, 3* | ENSDARG00000060127 | ENSDARP00000143433 | |
| *npffr2a* | *Neuropeptide FF receptor 2a* | ENSDARG00000033635 | ENSDARP00000088415 | |
| *gc* | *Gc, vitamin D binding protein* | ENSDARG00000089310 | ENSDARP00000140156 | |
| *slc4a4a* | *Solute carrier family 4 (sodium bicarbonate cotransporter), member 4a* | ENSDARG00000013730 | ENSDARP00000041502 | |
