# Supplementary material for: Comparative Genomics Studies on the dmrt Gene Family in Fish
Source: Front Genet. 2020 Nov 12;11:563947. doi: 10.3389/fgene.2020.563947 (PMC7689362; doi:10.3389/fgene.2020.563947)
Supplement: Supplementary file 4 [file Table_4.DOCX]

**Table S4.** Regulatory elements examined in this study.

| **Name** | **Sequences** | **Length (bp)** |
| --- | --- | --- |
| BRE | GGGCGCC | 7 |
|  | CCACGCC | 7 |
| CAAT box | GGTCAATCT | 9 |
|  | GGCCAATCT | 9 |
| E box | CACGTG | 6 |
| GC box | GGGCGG | 6 |
| TATA box | TATAAAA | 7 |
|  | TATAAAT | 7 |
|  | TATATAA | 7 |
|  | TATATAT | 7 |
