# Supplementary material for: Comparative Genomics Studies on the dmrt Gene Family in Fish
Source: Front Genet. 2020 Nov 12;11:563947. doi: 10.3389/fgene.2020.563947 (PMC7689362; doi:10.3389/fgene.2020.563947)
Supplement: Supplementary file 5 [file Table_5.DOCX]

**Table S5**. Numbers and accession numbers of the known *dmrt* sequences in Protacanthopterygii.

| **Gene** | **Parameter** | ***Salmo salar*** | ***Salmo trutta*** | ***Oncorhynchus nerka*** | ***Oncorhynchus tshawytscha*** | ***Oncorhynchus kisutch*** |
| --- | --- | --- | --- | --- | --- | --- |
| ***dmrt1*** | Numbers | 1 | 1 | 1 | 1 | 1 |
|  | Accession No. | XP_014028246.1 | XP_029573978.1 | XP_029492710.1 | XP_024275191.1 | XP_020328683.1 |
| ***dmrt2*** | Numbers | 4 | 3 | 4 | 4 | 2 |
|  | Accession No. | XP_013999067.1;  XP_014028244.1;  XP_013978971.1;  NP_001133069.1; | XP_029619763.1;  XP_029563884.1;  XP_029606321.1; | XP_029503165.1;  XP_029499527.1;  XP_029489111.1;  XP_029542911.1; | XP_024270527.1;  XP_024270536.1;  XP_024264070.1;  XP_024293941.1; | XP_020317727.1;  XP_020344428.1;  XP_020348560.1;  XP_020353213.1; |
| ***dmrt3*** | Numbers | 2 | 2 | 2 | 2 | 2 |
|  | Accession No. | XP_013999084.1;  XP_014028245.1; | XP_029600361.1;  XP_029619761.1; | XP_029512830.1;  XP_029511642.1; | XP_024296309.1;  XP_024275029.1; | XP_020317669.1;  XP_020325728.1; |
| ***dmrt4*** | Numbers | 1 | 1 | 1 | 1 | 1 |
|  | Accession No. | XP_014061928.1; | XP_029615604.1; | XP_029510939.1; | XP_024229664.1; | XP_020326315.1; |
| ***dmrt5*** | Numbers | 2 | 2 | 2 | 2 | 2 |
|  | Accession No. | XP_014072108.1;  XP_014024964.1; | XP_029606948.1;  XP_029563111.1; | XP_029542870.1;  XP_029486670.1; | XP_024277380.1;  XP_024271770.1; | XP_020353156.1;  XP_020347582.1; |
